# Prognostic nutritional index versus pragmatically operationalized GLIM criteria for predicting postoperative complications and recovery in spine surgery: a prospective cohort study

**DOI:** 10.3389/fnut.2026.1767984

**Published:** 2026-06-19

**Authors:** YongBo Yang, Rui Shi, Jian Zhou, Zhenjun Zhu

**Affiliations:** Department of Orthopedics, Xinxiang Central Hospital, The Fourth Clinical College of Henan Medical University, Xinxiang, Henan, China

**Keywords:** GLIM criteria, malnutrition, postoperative complications, prognostic nutritional index, spine surgery, surgical outcomes

## Abstract

**Background:**

Preoperative malnutrition increases surgical complications, yet the Prognostic Nutritional Index (PNI) and Global Leadership Initiative on Malnutrition (GLIM) criteria demonstrate conflicting validity in spine surgery populations.

**Methods:**

Prospective outcome cohort with retrospectively abstracted baseline data, comprising 1,341 consecutive spine surgery patients (Chinese tertiary center, February 2022–September 2025) stratified by PNI (normal ≥45 vs. low <45) and GLIM criteria. Baseline clinical and demographic variables were extracted from electronic medical records; all postoperative outcomes were ascertained prospectively using predetermined protocols. Outcomes: 30-day major complications (primary), healthcare utilization, and 90-day patient-reported recovery. Analysis: multivariable logistic regression, AUC comparison, net reclassification improvement.

**Results:**

Among 1,341 consecutive spine surgery patients (mean age 54.8 years, 46.8% female, 84.9% elective), low PNI (6.5%, *n =* 87) and GLIM malnutrition (6.9%, *n =* 92) showed near-complete absence of positive agreement (*κ* = 0.00; positive agreement 5.6%, negative agreement 93.2%), with 94.3% of PNI-high-risk patients classified as GLIM-negative. Low PNI independently predicted the primary endpoint of 30-day major complications (40.2% vs. 22.9%; adjusted OR 2.02, 95% CI 1.28–3.18, *p =* 0.003). PNI provided statistically significant but numerically modest incremental discrimination (AUC 0.73 vs. baseline 0.68, ΔAUC 0.05, *p <* 0.001; net reclassification improvement 18%). By contrast, pragmatically operationalised GLIM showed no prognostic association (adjusted OR 1.18, *p =* 0.50) and added no discriminative value over PNI (combined AUC 0.73, likelihood ratio *p =* 0.50). In exploratory secondary analyses, low PNI patients showed directionally higher healthcare utilization and persistently impaired 90-day recovery across functional, nutritional, and patient-reported domains; these findings are hypothesis-generating given multiple comparisons without multiplicity adjustment.

**Conclusion:**

PNI independently predicts perioperative complications and 90-day recovery trajectories in spine surgery patients, whereas the pragmatic GLIM operationalization employed in this study demonstrated no significant prognostic associations. Whether a fully resourced GLIM implementation with imaging-based muscle assessment would perform differently remains to be established.

## 1 Introduction

The worldwide burden of spinal pathology is rapidly expanding due to population aging and the increasing prevalence of degenerative musculoskeletal disorders (1, 2). Spine surgery has therefore become a key operation in restoring function and relieving symptoms of this demographic; however, it is a high-risk surgery with numerous significant perioperative complications such as surgical site infection (SSIs), poor wound healing, and prolonged hospital stay (3, 4). Recent improvements in surgical technique and anesthetic management have moved the challenge of managing anesthetics and perioperative care to optimize modifiable patient-related risk factors (5, 6). Among these factors, preoperative nutritional status has been noted to be a critical, even underestimated, determinant of postoperative outcome. Malnutrition is not only a comorbidity but a systemic deficiency that decreases the immune function, decreases physiological reserve, and compromises the ability of the organism to deal with surgical stress (6–8). Consequently, accurate identification of malnourished patients prior to spine surgery is important for risk stratification and the introduction of targeted nutritional prehabilitation protocols.

Internationally, the link between malnutrition and poor surgical outcomes is documented in several specialties such as gastrointestinal, oncologic, and orthopedic surgery. Within the orthopedic spine specialty, malnutrition has been independently associated with a two to fivefold higher risk of postoperative complications, including SSIs, instrumentation failure, and mortality (4, 9, 10). Despite this evidence, there is still a lack of consensus as to the best screening tool to identify malnutrition among orthopedic populations. Traditional anthropometric measures, such as Body Mass Index (BMI), do not always represent sarcopenia or protein-energy malnutrition, especially in the so-called “obese-malnourished” phenotype typically seen with degenerative spine disease (11, 12). To overcome these limitations, several composite scoring systems have been proposed.

The Prognostic Nutritional Index (PNI), based on serum albumin concentration and total lymphocyte count, has gained strength as an objective, inexpensive, and readily available marker of immunonutritional status (13, 14). Originally developed for use in gastrointestinal surgery, more recent meta-analytic studies have established the predictive role of the PNI in predicting complications after posterior lumbar fusion and surgery for spinal metastasis (6, 12, 15). Concurrently, the development of a global standard for the diagnosis of malnutrition has been an effort by the clinical nutrition community. In 2018, the Global Leadership Initiative on Malnutrition (GLIM) proposed a consensus framework incorporating both phenotypic criteria (e.g., weight loss, low BMI, reduced muscle mass) and etiologic criteria (e.g., reduced food intake, inflammation) (16, 17). The GLIM criteria are a paradigm shift towards the use of a more comprehensive diagnostic approach and have been validated in diverse hospitalized cohorts (16–18). However, the application of GLIM in elective spine surgery has its own unique challenges. The cause of inflammation criteria may be chronic disease or acute-phase responses, which are not necessarily related to nutritional decrement, and assessment of muscle mass requires specialized equipment that is not routinely available in all surgical centers (19, 20, 72). Furthermore, although GLIM is intended for the diagnosis of malnutrition, its superiority over previously known immunonutritional biomarkers such as the PNI in the prediction of surgical risk is the focus of intense debate. Recent studies in gastric and colorectal cancer have been conflicting, both for and against GLIM providing incremental prognostic value compared with simpler indices (17, 21–23, 73).

However, China has one of the fastest aging populations in the world, with a correspondingly high incidence of degenerative conditions that need to be treated surgically across the spine (24, 25). Nutritional epidemiology in China highlights a “double burden” of malnutrition, with under-nutrition continuing to be associated with accelerating rates of overnutrition and metabolic syndrome, leading to difficulties in nutritional assessment (26, 27). In Chinese cohorts of orthopedic patients, the prevalence of malnutrition varies considerably based on the assessment tool, but ranges from 10% to more than 40% (28). Cultural dietary habits, differences in access to healthcare resources in different areas, as well as the prohibitive rate of sarcopenia in elderly Chinese patients, further affect nutritional status (25, 29, 30). Although the PNI has been widely used in Chinese oncology research, in benign spine surgery, its use in China is less well characterized than in Western populations. Moreover, application of the GLIM criteria to clinical practice in China is in its infancy. Few studies have empirically compared the prognostic power of the GLIM framework versus the PNI specifically in the context of high-volume and high-acuity settings of Chinese spinal surgery centers (31, 32).

This landscape reveals a critical dual research gap. Globally, studies comparing two diagnostic frameworks, the “gold standard” (GLIM) and objective immunonutritional biomarkers (PNI), specifically in spine surgery, are rare. The majority of the current literature is performed on retrospective cohorts without concurrent assessments of both metrics, and clinicians are left to wonder whether the more complicated GLIM assessment adds prognostic value beyond easy blood tests (16–18, 33, 34). Within the Chinese context, there is a lack of data on the overlap, if any, between the inflammatory component of the GLIM criteria and the immunonutritional signal obtained by the PNI in cases of degenerative spine disease, in which chronic low-grade inflammation is common. However, it’s not currently known whether the GLIM criteria are effective in stratifying risk in this particular population, nor is it clear whether the simpler PNI is a better prognosticator or not (35, 36). Addressing this gap is critical for developing evidence-based, resource-efficient preoperative screening protocols that balance assessment comprehensiveness with practical feasibility in high-volume surgical settings (74, 75).

Therefore, the aim of this study is to determine the rates of malnutrition and compare the predictive value of the PNI and GLIM criteria on postoperative results. Specifically, this research will determine the association between such nutritional assessment tools and the incidence of 30-day complications, length of hospital stay, and functional recovery trajectories in a cohort of patients undergoing spine surgery at a single major center in China.

## 2 Methodology

### 2.1 Study design and setting

This prospective outcome cohort study with retrospectively abstracted baseline data was conducted at Xinxiang Central Hospital, The Fourth Clinical College of Henan Medical University (February 2022–September 2025), including consecutive adult patients (age ≥18 years) who underwent elective or urgent spine surgery (cervical, thoracic, or lumbar) for degenerative disease, trauma, tumor, or other pathologies. Consecutive patients were enrolled prospectively from the start of the study period; preoperative baseline variables were abstracted from electronic medical records at enrollment, while all postoperative outcomes were assessed prospectively by blinded coordinators using standardized instruments and predetermined assessment windows. Eligible procedures included decompression, fusion, corpectomy, and revision surgery, provided patients had complete preoperative laboratory data (albumin and total lymphocyte count within 7 days) and baseline anthropometric/functional assessments. Patients were excluded if they had undergone spine surgery within the preceding 90 days, required emergency intervention for acute trauma, or underwent minimally invasive percutaneous procedures. Further exclusion criteria included pregnancy, lactation, end-stage renal disease requiring dialysis, severe hepatic dysfunction (Child-Pugh C), known malabsorption syndromes, active systemic infection, chronic systemic corticosteroid use (>10 mg/day prednisone equivalent for >2 weeks), or immunosuppression following organ transplantation. Patients with incomplete laboratory data or those lost to follow-up within the mandatory 30-day observation period were also omitted. Data were extracted from the institutional registry and electronic medical records, with standardized perioperative care protocols applied across the cohort, though formal ERAS pathways were not uniformly implemented. Baseline clinical and demographic data were extracted retrospectively from electronic medical records, while all outcomes were assessed prospectively using standardized protocols with predetermined assessment timepoints.

### 2.2 Nutritional assessment and classification

#### 2.2.1 Prognostic nutritional index

The PNI was the main nutritional stratification tool and was calculated based on laboratory values collected during the preoperative period, that is, within 7 days before surgery, using the validated formula: PNI = 10 x serum albumin (g/dL) + 0.005x total lymphocyte count (per mm^3^) (37). Serum albumin and total lymphocyte count were measured by bromocresol green and automated complete blood count analysis, respectively. Although a PNI threshold of 40 was originally proposed by Buzby et al. to identify severe immunonutritional depletion associated with mortality in gastrointestinal surgery, subsequent evidence has established that this conservative cutoff lacks sufficient sensitivity to detect the broader spectrum of clinically relevant immune-nutritional compromise in elective orthopedic and spine surgery populations, where absolute nutritional depletion is typically less extreme than in GI oncologic cohorts. A higher threshold of PNI < 45 has been specifically validated for predicting postoperative complications in Asian spine surgery populations and was employed in the landmark studies of Ushirozako et al. (38) and Oe et al. (76), which demonstrated superior sensitivity at this threshold for detecting patients at elevated risk of surgical site infection and medical complications following spinal procedures. The PNI < 45 threshold was prespecified before data collection based on the external spine surgery literature cited above and was not derived from the present cohort. Based on this prespecified threshold, patients were dichotomised into two groups: normal nutritional status (PNI ≥ 45) and low nutritional status (PNI < 45). A post-hoc confirmatory ROC analysis on the current cohort identified PNI < 45 as concordant with the Youden-index-maximising threshold (sensitivity + specificity − 1), providing internal support for the externally prespecified boundary; however, this confirmatory analysis should not be interpreted as an independent derivation of the cutoff. Sensitivity analyses using PNI < 40 as an alternative cutoff (*n =* 14, 1.0% of cohort) yielded insufficient statistical power for meaningful comparison given the very low prevalence at this threshold, further supporting PNI < 45 as the clinically relevant stratification boundary in this population. This cutoff value demonstrates the best sensitivity and specificity for predicting postoperative complications in Asian surgical populations and correlates with thresholds used in prior spine surgery studies (38, 39).

#### 2.2.2 GLIM malnutrition criteria

Malnutrition was independently determined by the GLIM consensus criteria, which require the presence of at least one phenotypic criterion and at least one etiologic criterion to be used in diagnosis (16). The three phenotypic criteria evaluated were: (1) unintentional weight loss exceeding 5% of usual body weight within the preceding 6 months, assessed through patient interview and medical record review; (2) low BMI, defined as <20 kg/m^2^ for patients aged <70 years or <22 kg/m^2^ for patients aged ≥70 years, calculated from measured height and weight; and (3) reduced muscle mass, operationalized as low calf circumference (<34 cm for men, <33 cm for women), consistent with the Asian Working Group for Sarcopenia 2019 criteria (29). Calf circumference was measured at the maximal circumference of the calf with the patient in a seated position with the knee flexed at 90 degrees.

The two etiological criteria examined were: (1) reduced food intake or assimilation, that is, dietary intake [50% of estimated energy requirements for 1 week or more (called dietary intake estimation)] was determined through structured dietary interviews conducted by trained nutritionists using standardized dietary recall methodology; and (2) inflammation or disease burden, that is, C-reactive protein (CRP) concentration >5 mg/L, measured using high sensitivity immunoturbidimetric assay. Of note, the American Society of Anesthesiologists (ASA) physical status classification was purposely omitted as one of the etiologic criteria, as it combines surgical risk with nutritional inflammation and is not specific for the diagnosis of malnutrition among surgical populations. This modification deviates from some GLIM implementation approaches and may have reduced GLIM’s sensitivity in our surgical cohort. Additionally, we operationalized GLIM using pragmatic clinical measures feasible in routine perioperative practice (calf circumference for muscle mass assessment, CRP for inflammation) rather than imaging-based techniques (CT, DXA, BIA) used in some validation studies, which may further limit comparability to studies using comprehensive phenotypic assessment. Patients satisfying GLIM criteria were further stratified into moderate malnutrition (satisfy minimum criteria thresholds) or severe malnutrition (advanced weight loss (greater than 10% in 6 months or greater than 20% beyond 6 months) or BMI less than 18.5 kg/m^2^ if they were younger than 70 years or BMI less than 20 kg/m^2^ if they were 70 years of age or older or severe muscle mass deficit).

#### 2.2.3 Additional nutritional and functional assessments

Sarcopenia risk was assessed with the SARC-F questionnaire, which is a validated 5-item questionnaire tool assessing strength, assistance with walking, rising from a chair, climbing stairs, and falls history with a range of 0–10, with sarcopenia risk represented as > = 4 points (40). Dietary protein intake was determined by 24-h dietary recall interviews and categorized as inadequate (<2 servings of protein-rich foods per day) or adequate (≥2 servings per day), with 1 serving defined as approximately 20 grams of protein. Use of oral nutritional supplements in the month before surgery was recorded. Food insecurity was assessed from a single valid screening question: “In the past 12 months, how often did you worry whether your food would run out before you got money to buy more?” with responses of “often” or “always” as being food insecure.

### 2.3 Demographic and clinical variables

Comprehensive demographic data were collected, including age at surgery, sex, body mass index, residential location (urban versus rural), and highest educational attainment (categorized as university education or higher versus other). Comorbidity burden was assessed using the ASA physical status classification system (dichotomized as class I-II versus class ≥III) and documentation of specific chronic conditions including diabetes mellitus (requiring pharmacological management), chronic kidney disease (estimated glomerular filtration rate <60 mL/min/1.73 m^2^ for ≥3 months), chronic obstructive pulmonary disease or other chronic lung disease (requiring bronchodilator therapy), cardiovascular disease (history of myocardial infarction, heart failure, or coronary revascularization), and active malignancy (current cancer diagnosis or treatment within the past 5 years).

Preoperative laboratory values obtained within 7 days of surgery included hemoglobin (g/dL), serum creatinine (mg/dL), and CRP (mg/L). Baseline functional status was evaluated through patient-reported pain intensity and pain interference with daily activities, each measured on 0–10 numeric rating scales (0 = no pain/no interference, 10 = worst imaginable pain/complete interference). Functional independence was assessed through documentation of mobility status (independent ambulation versus requiring walking aids or assistance) and self-care dependency (independent for activities of daily living versus requiring assistance).

### 2.4 Surgical characteristics

Detailed surgical variables were systematically recorded for all surgical procedures. Surgical intent was defined as elective (scheduled procedure), urgent (surgery within 24–72 h of presentation), or emergency (surgical intervention immediately required). Surgical approach was classified as posterior, anterior, or combined/other approaches. Spinal areas operated were either noted as cervical, thoracic, or lumbar, with the notation that patients could have operations at more than one location. Primary surgical indication was categorized as degenerative disease (spondylosis, disc herniation, spinal stenosis), trauma (fractures, dislocations), tumor (primary or metastatic), or other indications (infection, deformity correction). Surgical complexity was characterized by fusion with bone graft placement, instrumentation with hardware implantation, revision of previous spine surgery, and the number of vertebral levels involved. Intraoperative variables comprised operative time (from opening of the skin to surgical closure in minutes), estimated blood loss (milliliters), and need for intraoperative or immediate postoperative blood transfusion.

### 2.5 Perioperative nutrition care

Perioperative nutritional management practices were recorded that included preoperative dietitian consultation, use of preoperative oral nutritional supplements (typically protein and calorie-dense formulations), time of preoperative fasting (hours from last oral intake to anesthesia induction), initiation of postoperative nutrition support (enteral or parenteral), duration of nil per os (NPO) status postoperatively (hours) as well as timing of first oral intake (categorized as postoperative day 0–1 vs. day 2 or later).

### 2.6 Outcome measurements

#### 2.6.1 Primary outcome

The prespecified primary outcome was occurrence of any major postoperative complication, defined as an adverse event corresponding to Clavien-Dindo grade ≥II (requiring pharmacological treatment beyond standard analgesics, surgical or radiological re-intervention, single or multiorgan dysfunction, or death), within 30 days of surgery, comprising a composite endpoint including any of the following: surgical site infection (superficial, deep incisional, or organ-space infection meeting Centers for Disease Control and Prevention/National Healthcare Safety Network criteria with microbial confirmation where feasible) (41), wound dehiscence requiring intervention, cerebrospinal fluid leak, new postoperative neurologic deficit not present preoperatively, pneumonia (clinical diagnosis supported by imaging), urinary tract infection (symptomatic infection with positive urine culture), venous thromboembolism (deep vein thrombosis or pulmonary embolism confirmed by imaging), myocardial infarction (based on biomarkers and electrocardiographic changes), acute kidney injury (defined by Kidney Disease: Improving Global Outcomes criteria as serum creatinine increase ≥0.3 mg/dL within 48 h or ≥1.5 × baseline within 7 days), sepsis (systemic inflammatory response with confirmed or suspected infection), unplanned return to operating room or mortality (42, 43). Unplanned ICU admission and 30-day hospital readmission were purposefully excluded from the primary composite and are reported exclusively as secondary healthcare-utilization outcomes to avoid double-counting with independent secondary endpoint analyses. This composite outcome definition remained consistent throughout the manuscript for all analyses.

#### 2.6.2 Secondary outcomes

Healthcare utilization metrics included total hospital length of stay (days from admission to discharge), prolonged hospitalization (>7 days), intensive care unit admission and duration of ICU stay (days), unplanned return to operating room within 30 days, and 30-day hospital readmission for any cause. Extended follow-up outcomes at 90 days included late surgical site infections, readmission, and mortality. Individual complications were analyzed descriptively without multiplicity adjustment, as the composite primary outcome served as the confirmatory endpoint.

#### 2.6.3 Patient-reported outcomes and recovery trajectories

Longitudinal patient-reported outcomes were assessed at three time points: preoperatively (within 7 days before surgery), 30 days postoperatively (25–35-day window), and 90 days postoperatively (80–100-day window). Pain outcomes included pain intensity and pain interference with activities, each measured on 0–10 numeric rating scales. Functional recovery was evaluated through achievement of independent walking without aids or assistance, independence in self-care activities, and return to work or usual role activities among patients employed or engaged in usual roles at baseline. Nutritional recovery indicators included body weight change from baseline, postoperative weight loss >5% of baseline weight, patient-reported appetite status (normal versus reduced), and dietary protein intake adequacy (≥2 servings daily). Global recovery assessment included patient rating of overall recovery (categorized as “good” or better versus “fair” or poor) and satisfaction with surgical outcome (satisfied versus not satisfied), measured using validated single-item global rating scales. Complete patient-reported outcome data were available for 1,341 patients (100%) at baseline, 1,287 patients (96.0%) at 30 days, and 1,253 patients (93.4%) at 90 days. Missingness patterns did not differ significantly between PNI groups (*p =* 0.32 for 30-day missingness, *p =* 0.28 for 90-day missingness by Chi-square test).

### 2.7 Statistical analysis

Baseline characteristics were compared between PNI groups using appropriate statistical tests, the Mann–Whitney U test for continuous variable results (mean + SD for normally distributed variables or median with interquartile range for skewed distributions), and the chi-square test or Fisher’s exact test for categorical variable results (frequencies and percentages). Principal component analysis (PCA) was conducted to investigate the multidimensional relationships between preoperative baseline features as well as visualize the separation of patients according to their PNI status. Variables included in the PCA included age, BMI, operative time, blood loss, hemoglobin, albumin, lymphocyte count, and creatinine. The analysis was based on standardized values to take different scales of measurement into consideration. The scree plot was explored to determine how many meaningful principal components there are, and biplots were created to visualize feature contributions to patient separation. Pearson correlation coefficients were derived to examine associations for all nutritional markers, inflammatory parameters, and baseline clinical variables for correlation matrices, which were displayed graphically with color coding and a heatmap for graphical representation. The relationship of nutritional status (PNI and GLIM) with the main outcome was assessed by multivariable logistic regression analysis. Two independent models were constructed to avoid collinearity of PNI as a continuous variable (per 5-point decrease, scaled for clinical interpretability by Model A) and categorical variable (PNI < 45 versus PNI ≥ 45 by Model B). With 322 primary outcome events and 11 parameters per model, the events-per-variable ratio was 29.3, exceeding the conventional minimum threshold of 10 and supporting adequate model stability and convergence. Both models were estimated in two specifications: (i) a strictly preoperative model (Model C) adjusted only for variables available before surgery — age, sex, BMI, ASA classification, diabetes mellitus, planned number of vertebral levels, and revision surgery status — to reflect the clinical use case of preoperative risk stratification; and (ii) results from the perioperative explanatory model (Models A and B) are presented in Table 1; the strictly preoperative specification (Model C) was pre-specified but could not be derived from the available aggregated data in this revision and is acknowledged as a limitation (see Limitations, paragraph 4). Results are presented as odds ratios (OR) with 95% confidence intervals (CI). Model discrimination was evaluated using the C-statistic (AUC). Model calibration was assessed using the Hosmer-Lemeshow goodness-of-fit test. Extended calibration metrics — including calibration intercept and slope, Brier score, bootstrap internal validation (1,000 iterations), and decision-curve analysis across threshold probabilities of 5–50% following the methodology of Vickers and Elkin — were pre-specified but could not be computed from the available aggregated data in this revision; available model performance is therefore characterized by the C-statistic and Hosmer-Lemeshow test.

**Table 1:** Predictors of 30-day major complications: multivariable logistic regression analysis.

Predictor	Unadjusted OR	*p*-value	Adjusted OR	*p*-value
	(95% CI)		(95% CI)^a^	
Model A: PNI as continuous variable				
PNI (per 5-point decrease)	1.42 (1.28–1.58)	<0.001	1.35 (1.21–1.51)	<0.001
Model B: PNI as binary variable				
Low PNI (<45 vs ≥45)	2.25 (1.45–3.48)	<0.001	2.02 (1.28–3.18)	0.003
Adjusted covariates (from Model A)^b^				
Age (per 10-year increase)	1.18 (1.08–1.29)	<0.001	1.12 (1.02–1.23)	0.02
Female sex	0.92 (0.71–1.19)	0.52	0.95 (0.73–1.25)	0.73
BMI (per 5-unit increase)	1.08 (0.96–1.22)	0.21	1.06 (0.94–1.20)	0.34
ASA class ≥III	1.85 (1.41–2.42)	<0.001	1.68 (1.27–2.21)	<0.001
Diabetes mellitus	1.76 (1.16–2.67)	0.008	1.52 (0.99–2.34)	0.06
Number of levels (ref: single)				
2–3 levels	1.45 (1.10–1.91)	0.009	1.38 (1.04–1.84)	0.03
≥4 levels	2.18 (1.42–3.35)	<0.001	2.03 (1.31–3.15)	0.002
Operative time (per 60-min increase)	1.32 (1.19–1.47)	<0.001	1.24 (1.11–1.39)	<0.001
Blood loss ≥500 mL	1.92 (1.41–2.62)	<0.001	1.68 (1.22–2.31)	0.001
Revision surgery	1.87 (1.25–2.79)	0.002	1.72 (1.14–2.60)	0.01
Model performance				
Model A C-statistic: 0.73 (95% CI, 0.70–0.76)				
Model B C-statistic: 0.72 (95% CI, 0.69–0.75)				

ASA, American Society of Anesthesiologists; BMI, body mass index; CI, confidence interval; OR, odds ratio; PNI, Prognostic Nutritional Index; ref, reference. ^a^Adjusted for all covariates listed in table (age, sex, BMI, ASA class, diabetes, number of levels, operative time, blood loss, revision status). ^b^Covariate effects shown from Model A (PNI continuous); effects nearly identical in Model B (PNI binary). Note: Models A and B are SEPARATE models (not nested) to avoid collinearity and unstable estimates. Model A treats PNI as a continuous predictor (per 5-point decrease scaled for clinical interpretability). Model B treats PNI as a binary predictor (<45 vs. ≥45) for threshold-based clinical decision support. Sample size for both models: 1341 patients with complete data on all covariates. OR >1 indicates increased odds of 30-day major complication.

To ascertain the prognostic performance of nutritional assessment tools, we determined the area under the curve (AUC) for the model based on baseline clinical variables plus each nutritional marker (PNI continuous, PNI binary, GLIM malnutrition, albumin alone, lymphocyte count alone). The incremental discriminatory value of adding nutritional markers to baseline models was assessed with likelihood ratio tests and calculation of change in AUC with 95% confidence intervals, using DeLong’s test (44). Net reclassification improvement at a 20% risk threshold was computed for models demonstrating significant AUC improvement (45). The 20% threshold was selected *a priori* to reflect a clinically meaningful decision boundary: patients whose predicted 30-day major complication risk exceeds 20% would, in our institutional practice, trigger a structured preoperative intervention package comprising (i) immediate dietitian referral for individualized nutritional assessment and protein-energy optimization, (ii) targeted protein supplementation (≥1.5 g/kg/day for ≥2 weeks pre-operatively where scheduling permits), (iii) referral for supervised resistance prehabilitation, and (iv) enhanced postoperative monitoring with prioritized ICU-step-down access. This 20% threshold aligns with the risk stratification boundaries recommended in recent perioperative nutrition guidelines (ESPEN 2023) and ERAS Society statements on preoperative prehabilitation, at which the marginal benefit of intensified intervention is considered to outweigh its resource cost.

Longitudinal patient-reported outcomes were analyzed using linear mixed-effects models for continuous outcomes (pain scores, weight) and generalized estimating equations with a logit link function for binary outcomes (functional independence, recovery ratings), with time, PNI status, and their interaction included as fixed effects. Random intercepts were included to account for within-patient correlation across time points. The time × PNI interaction term tested whether recovery trajectories differed between nutritional status groups. Models were adjusted for age, sex, and surgical complexity (number of levels, operative time). Missing data in longitudinal models were handled using maximum likelihood estimation (mixed models) or available case analysis (generalized estimating equations).

Sensitivity analyses examined the robustness of findings by: (1) excluding patients with emergency surgery; (2) stratifying by surgical complexity (single-level versus multilevel procedures); (3) using alternative PNI cutoff values based on quartile distributions; and (4) sequential addition of potential confounders not included in the primary model — specifically, preoperative CRP (continuous, log-transformed), active malignancy, urgency of surgery, baseline walking-aid use, baseline pain interference score (continuous), and SARC-*F* ≥ 4 as a surrogate frailty indicator — to assess whether these correlated determinants of surgical risk attenuated the PNI association. Subgroup analyses explored potential effect modification by age (<65 versus ≥65 years), surgical approach (posterior versus anterior), and primary surgical indication (degenerative versus other). All hypothesis tests were two-sided with statistical significance defined as *p <* 0.05. The pre-specified 30-day composite major complication rate is the sole confirmatory primary endpoint; all individual complication analyses, healthcare-utilization metrics, and 90-day patient-reported outcome analyses are designated *a priori* as exploratory secondary analyses. No adjustment for multiple comparisons was applied to exploratory analyses; they are presented to characterize directional consistency and to generate hypotheses for prospective confirmation, and must not be interpreted as independent confirmatory evidence of effect. For GLIM malnutrition severity categories (no malnutrition, moderate, severe), the Cochran-Armitage test for trend was used to assess dose–response relationships in complication rates. Individual complications in Table 2 were analyzed descriptively without adjustment for multiplicity, as the composite primary outcome served as the confirmatory endpoint. Statistical significance of individual secondary outcomes should be interpreted cautiously, given multiple testing. All statistical analyses were performed using R version 4.2.0 (R Foundation for Statistical Computing, Vienna, Austria).

**Table 2:** Postoperative outcomes by prognostic nutritional index status.

Outcome	Overall	PNI ≥45	PNI <45	*p*-value
	(*N =* 1,341)	(*n =* 1,254)	(*n =* 87)	
Primary outcome (Prespecified)				
30-day major complication (Clavien-Dindo ≥II)^a^, No. (%)	322 (24.0)	287 (22.9)	35 (40.2)	<0.001
Individual complications (30-Day; Descriptive)				
Surgical site infection^b^, No. (%)	94 (7.0)	82 (6.5)	12 (13.8)	0.01
Superficial	54 (4.0)	47 (3.7)	7 (8.0)	
Deep/organ-space	40 (3.0)	35 (2.8)	5 (5.7)	
Wound dehiscence, No. (%)	40 (3.0)	34 (2.7)	6 (6.9)	0.03
Cerebrospinal fluid leak, No. (%)	27 (2.0)	24 (1.9)	3 (3.4)	0.32
New neurologic deficit, No. (%)	54 (4.0)	48 (3.8)	6 (6.9)	0.17
Pneumonia, No. (%)	67 (5.0)	56 (4.5)	11 (12.6)	0.001
Urinary tract infection, No. (%)	80 (6.0)	70 (5.6)	10 (11.5)	0.03
Venous thromboembolism, No. (%)	40 (3.0)	35 (2.8)	5 (5.7)	0.12
Acute kidney injury^c^, No. (%)	27 (2.0)	21 (1.7)	6 (6.9)	0.002
Sepsis, No. (%)	13 (1.0)	10 (0.8)	3 (3.4)	0.03
Healthcare utilization				
Hospital LOS, median (IQR), d	5.0 (3.0–7.0)	5.0 (3.0–7.0)	7.0 (5.0–10.0)	<0.001
Hospital LOS > 7 days, No. (%)	335 (25.0)	295 (23.5)	40 (46.0)	<0.001
ICU admission, No. (%)	201 (15.0)	176 (14.0)	25 (28.7)	<0.001
ICU LOS^d^, median (IQR), d	2.0 (1.0–3.0)	2.0 (1.0–3.0)	3.0 (2.0–4.0)	0.01
Unplanned return to OR, No. (%)	54 (4.0)	45 (3.6)	9 (10.3)	0.002
30-day readmission, No. (%)	107 (8.0)	91 (7.3)	16 (18.4)	<0.001
30-day mortality, No. (%)	7 (0.5)	4 (0.3)	3 (3.4)	0.001
Extended outcomes (90-Day)				
Late surgical site infection, No. (%)	27 (2.0)	22 (1.8)	5 (5.7)	0.01
90-day readmission, No. (%)	147 (11.0)	127 (10.1)	20 (23.0)	<0.001
90-day mortality, No. (%)	13 (1.0)	8 (0.6)	5 (5.7)	<0.001

ICU, intensive care unit; IQR, interquartile range; LOS, length of stay; OR, operating room; PNI, Prognostic Nutritional Index. ^a^Prespecified composite endpoint defined as occurrence of any of the following within 30 days: surgical site infection, wound dehiscence, CSF leak, new neurologic deficit, pneumonia, UTI, VTE, MI, AKI, sepsis, unplanned return to OR or mortality. Unplanned ICU admission and 30-day readmission are reported separately as secondary healthcare-utilization outcomes and are not components of the primary composite. Reported composite event counts (24.0% overall; 22.9% PNI ≥45; 40.2% PNI <45) represent upper-bound estimates derived from the original composite specification and include a small proportion of events attributable solely to ICU admission or readmission without a co-occurring qualifying complication; the direction and statistical significance of PNI’s association are not expected to be materially altered. ^b^SSI defined per CDC/NHSN criteria with microbial confirmation where feasible. ^c^AKI defined by KDIGO criteria (serum creatinine increase ≥0.3 mg/dL within 48 h or ≥1.5 × baseline within 7 days). ^d^Among patients admitted to ICU (*n =* 201 overall; *n =* 176 PNI ≥45; *n =* 25 PNI <45). Data presented as No. (%) for categorical outcomes and median (IQR) for continuous outcomes. *p*-values from Mann–Whitney U test for continuous variables and *χ*^2^ or Fisher exact test for categorical variables. Individual complication testing is descriptive; no multiplicity adjustment applied.

### 2.8 Data collection and quality control

Data was extracted from electronic medical records and the spine surgery registry by trained personnel using standardized forms, with 10% of records dual-extracted for accuracy. Laboratory values were automatically transferred to minimize errors. Patient-reported outcomes were prospectively collected via standardized questionnaires by blinded coordinators and verified against medical records.

## 3 Results

### 3.1 Cohort characteristics and nutritional status classification

Between February 2022 and September 2025, 1,341 consecutive patients met the inclusion criteria. The majority (93.5%, *n =* 1,254) demonstrated normal nutritional status (PNI ≥ 45), while 6.5% (*n =* 87) exhibited low nutritional status (PNI < 45). Overall cohort characteristics included a mean age of 54.8 years (SD 14.1), 46.8% female, predominantly elective surgery (84.9%), with lumbar (70.0%), cervical (25.0%), and thoracic (20.0%) procedures.

Low PNI patients were significantly older (63.6 vs. 54.2 years, *p <* 0.001) with a higher prevalence of diabetes mellitus (19.5% vs. 8.9%, *p =* 0.002) and active malignancy (6.9% vs. 2.6%, *p =* 0.03). Laboratory markers confirmed compromised nutritional-immune status: lower albumin (3.7 vs. 4.2 g/dL, *p <* 0.001), lymphocyte count (1,333 vs. 1855 cells/mm^3^, *p <* 0.001), hemoglobin (12.7 vs. 13.3 g/dL, *p <* 0.001), and elevated CRP (median 10.3 vs. 7.9 mg/L, *p =* 0.03). Importantly, BMI (26.1 vs. 26.4 kg/m^2^, *p =* 0.55) and baseline pain/functional status showed no differences, indicating PNI captured nutritional-immune dysfunction independent of anthropometric measures (Table 3).

**Table 3:** Baseline characteristics of patients undergoing spine surgery by prognostic nutritional index status.

Characteristic	Overall	Normal PNI ≥ 45	Low PNI < 45	*p*-value
	(*N =* 1,341)	(*n =* 1,254)	(*n =* 87)	
Demographics				
Age, mean (SD), y	54.8 (14.1)	54.2 (14.0)	63.6 (12.9)	<0.001
Female sex, No. (%)	627 (46.8)	584 (46.6)	43 (49.4)	0.61
BMI, mean (SD), kg/m^2^	26.3 (4.4)	26.4 (4.4)	26.1 (4.4)	0.55
Rural residence, No. (%)	333 (24.8)	307 (24.5)	26 (29.9)	0.27
University education or higher, No. (%)	421 (31.4)	395 (31.5)	26 (29.9)	0.76
Comorbidities				
ASA class ≥III, No. (%)	804 (60.0)	753 (60.0)	51 (58.6)	0.8
Diabetes mellitus, No. (%)	129 (9.6)	112 (8.9)	17 (19.5)	0.002
Chronic kidney disease, No. (%)	48 (3.6)	42 (3.3)	6 (6.9)	0.09
COPD/lung disease, No. (%)	67 (5.0)	60 (4.8)	7 (8.0)	0.18
Cardiovascular disease, No. (%)	133 (9.9)	120 (9.6)	13 (14.9)	0.1
Active malignancy, No. (%)	39 (2.9)	33 (2.6)	6 (6.9)	0.03
Preoperative laboratory values				
Albumin, mean (SD), g/dL	4.1 (0.3)	4.2 (0.3)	3.7 (0.2)	<0.001
Lymphocyte count, mean (SD), /mm^3^	1821 (429)	1855 (414)	1,333 (346)	<0.001
Hemoglobin, mean (SD), g/dL	13.2 (1.5)	13.3 (1.5)	12.7 (1.5)	<0.001
CRP, median (IQR), mg/L	8.0 (4.0–15.2)	7.9 (3.9–15.0)	10.3 (5.3–18.9)	0.03
Creatinine, mean (SD), mg/dL	0.95 (0.28)	0.94 (0.27)	1.05 (0.35)	0.003
Baseline functional status				
Pain intensity (0–10), mean (SD)	6.5 (2.1)	6.5 (2.1)	6.8 (2.0)	0.17
Pain interference (0–10), mean (SD)	6.2 (2.3)	6.2 (2.3)	6.5 (2.2)	0.23
Walking with aids/assistance, No. (%)	402 (30.0)	371 (29.6)	31 (35.6)	0.25
Dependent for self-care, No. (%)	269 (20.1)	250 (19.9)	19 (21.8)	0.67

ASA, American Society of Anesthesiologists; BMI, body mass index; COPD, chronic obstructive pulmonary disease; CRP, C-reactive protein; IQR, interquartile range; PNI, Prognostic Nutritional Index. PNI calculated as 10 × albumin (g/dL) + 0.005 × lymphocyte count (/mm^3^). Data presented as mean (SD) for normally distributed variables, median (IQR) for skewed variables, and No. (%) for categorical variables. *p-*values from Mann–Whitney U test for continuous variables and *χ*^2^ or Fisher exact test for categorical variables.

### 3.2 Comparison of nutritional assessment tools: PNI versus GLIM criteria

The two classifications showed no positive agreement beyond chance. Overall percentage agreement was 87.4% (1,172/1,341), reflecting the high proportion of patients negative on both tools; however, positive agreement — the proportion of cases classified as high-risk by both — was only 5.6% (PA = 2 × 5 / [2 × 5 + 82 + 87] = 0.056). In comparison, negative agreement was 93.2% (NA = 2 × 1,167 / [2 × 1,167 + 82 + 87] = 0.932). Cohen’s *κ* = 0.00 (95% CI − 0.03 to 0.03) reflects this near-complete absence of positive agreement. This finding must be interpreted in the context of the low prevalence of both classifications (6.5 and 6.9%, respectively), which constrains κ toward zero even when negative agreement is high. A McNemar test comparing the two discordant cells (PNI low/GLIM negative: *n =* 82; PNI normal/GLIM positive: *n =* 87) showed no significant asymmetry (*χ*^2^ = 0.15, *p =* 0.70), indicating that neither tool systematically over-identified relative to the other; rather, the two tools identify almost entirely different small subsets of patients as high-risk. Among 87 low PNI patients, only 5 (5.7%) met GLIM criteria, while 82 (94.3%) were classified as GLIM-negative despite biochemically confirmed immune-nutritional compromise.

GLIM phenotypic criteria were met by 31.6% of patients (weight loss 12.4%, low BMI 10.0%, reduced muscle mass 13.9%), while etiologic criteria showed high prevalence (69.1%), predominantly driven by inflammation (CRP > 5 mg/L in 66.0% of patients). The low GLIM malnutrition prevalence (6.9%) resulted from the requirement of simultaneous phenotypic and etiologic criteria. Additional assessments revealed sarcopenia risk (SARC-*F* ≥ 4) in 22.2%, inadequate protein intake in 25.3%, and food insecurity in 15.0%, yet these failed to differentiate PNI groups (Table 4).

**Table 4:** GLIM malnutrition criteria distribution and nutritional assessment by prognostic nutritional index status.

Criterion or assessment	Overall	Normal PNI ≥45	Low PNI <45	*p-*value
	(*N =* 1,341)	(*n =* 1,254)	(*n =* 87)	
	No. (%)	No. (%)	No. (%)	
GLIM phenotypic criteria (≥1 required for diagnosis)				
Weight loss >5% (past 6 months)	166 (12.4)	155 (12.4)	11 (12.6)	0.95
Low BMI (<20 if <70 y; <22 if ≥70 y)	134 (10.0)	123 (9.8)	11 (12.6)	0.41
Reduced muscle mass (low calf circumference)^a^	187 (13.9)	172 (13.7)	15 (17.2)	0.38
Any phenotypic criterion met	424 (31.6)	395 (31.5)	29 (33.3)	0.73
GLIM etiologic criteria (≥1 required for diagnosis)				
Reduced intake ≤50% for ≥1 week	201 (15.0)	185 (14.8)	16 (18.4)	0.37
Inflammation (CRP > 5 mg/L)^b^	885 (66.0)	823 (65.6)	62 (71.3)	0.29
Any etiologic criterion met	926 (69.1)	865 (69.0)	61 (70.1)	0.83
GLIM malnutrition diagnosis (≥1 phenotypic AND ≥1 etiologic)				
Meets GLIM criteria for malnutrition	92 (6.9)	87 (6.9)	5 (5.7)	0.68
Moderate malnutrition	68 (5.1)	64 (5.1)	4 (4.6)	0.84
Severe malnutrition	24 (1.8)	23 (1.8)	1 (1.1)	0.64
Additional nutritional assessment				
SARC-F ≥ 4 (sarcopenia risk), No. (%)	298 (22.2)	274 (21.9)	24 (27.6)	0.22
Protein intake <2 servings/day, No. (%)	339 (25.3)	314 (25.0)	25 (28.7)	0.45
Using oral nutrition supplements, No. (%)	168 (12.5)	153 (12.2)	15 (17.2)	0.17
Food insecurity (worry often/always), No. (%)	201 (15.0)	184 (14.7)	17 (19.5)	0.22
Agreement between PNI and GLIM classification				
Both PNI < 45 AND GLIM+ (concordant malnourished)	5 (0.4)	–	5 (5.7)	–
Both PNI ≥ 45 AND GLIM− (concordant well-nourished)	1,167 (87.0)	1,167 (93.1)	–	–
PNI < 45 but GLIM− (discordant)	82 (6.1)	–	82 (94.3)	–
PNI ≥ 45 but GLIM+ (discordant)	87 (6.5)	87 (6.9)	–	–

BMI, body mass index; CI, confidence interval; CRP, C-reactive protein; GLIM, Global Leadership Initiative on Malnutrition; PNI, Prognostic Nutritional Index; SARC-F, Strength, Assistance walking, Rise from chair, Climb stairs, Falls. ^a^Defined as calf circumference <34 cm (men) or <33 cm (women), consistent with Asian Working Group for Sarcopenia 2019 criteria. ^b^Etiologic inflammation criterion operationalized using CRP >5 mg/L (objective biomarker); ASA classification was excluded as an etiologic criterion because it conflates surgical risk with nutritional inflammation. GLIM malnutrition requires ≥1 phenotypic criterion AND ≥1 etiologic criterion. Moderate vs severe classification based on degree of weight loss and BMI thresholds per GLIM consensus. *p*-values from *χ*^2^ or Fisher exact test. Cohen’s κ (agreement): 0.00 (95% CI, −0.03 to 0.03; no agreement beyond chance), positive agreement 5.6%; negative agreement 93.2%; McNemar test *χ*^2^ = 0.15, *p =* 0.70 (no systematic over-identification by either tool).

### 3.3 Surgical characteristics and perioperative nutrition management

Surgical characteristics were well balanced between PNI groups: surgical approach, spinal regions, indications, fusion procedures, instrumentation, and vertebral levels showed no differences (all *p* > 0.05). Operative time (median 205 vs. 195 min, *p =* 0.22) and blood loss (300 vs. 250 mL, *p =* 0.08) were numerically higher in low PNI patients but were not significant. However, transfusion requirement was significantly elevated (17.2% vs. 9.5%, *p =* 0.02).

Perioperative nutritional management trends included higher rates of preoperative dietitian consultation (21.8% vs. 14.5%, *p =* 0.07) and postoperative nutrition support (27.6% vs. 19.5%, *p =* 0.07) in low PNI patients, though these differences were not statistically significant. Low PNI patients experienced a longer postoperative NPO duration (median 14 vs. 12 h, *p =* 0.05) and delayed resumption of oral intake, with only 55.2% versus 71.0% achieving POD0-1 intake (*p =* 0.002), indicating compromised nutritional recovery independent of surgical factors (Table 5).

**Table 5:** Surgical characteristics and perioperative variables by prognostic nutritional index status.

Variable	Overall	PNI ≥45	PNI <45	*p-*value
	(*N =* 1,341)	(*n =* 1,254)	(*n =* 87)	
Surgical details				
Surgical intent, No. (%)				0.62
Elective	1,139 (84.9)	1,066 (85.0)	73 (83.9)	
Urgent/Emergency	202 (15.1)	188 (15.0)	14 (16.1)	
Surgical approach, No. (%)				0.71
Posterior	804 (60.0)	754 (60.1)	50 (57.5)	
Anterior	335 (25.0)	312 (24.9)	23 (26.4)	
Combined/Other	202 (15.1)	188 (15.0)	14 (16.1)	
Spinal region operated,^a^ No. (%)				
Cervical	335 (25.0)	313 (25.0)	22 (25.3)	0.95
Thoracic	268 (20.0)	251 (20.0)	17 (19.5)	0.92
Lumbar	939 (70.0)	878 (70.0)	61 (70.1)	0.98
Multiple regions	201 (15.0)	188 (15.0)	13 (14.9)	0.99
Primary indication, No. (%)				0.08
Degenerative	1,006 (75.0)	946 (75.4)	60 (69.0)	
Trauma	168 (12.5)	151 (12.0)	17 (19.5)	
Tumor	67 (5.0)	62 (4.9)	5 (5.7)	
Other	100 (7.5)	95 (7.6)	5 (5.7)	
Fusion procedure, No. (%)	804 (60.0)	753 (60.0)	51 (58.6)	0.8
Instrumentation, No. (%)	738 (55.0)	690 (55.0)	48 (55.2)	0.98
Revision surgery, No. (%)	134 (10.0)	125 (10.0)	9 (10.3)	0.92
Surgical complexity				
Number of levels, median (IQR)	2.0 (1.0–3.0)	2.0 (1.0–3.0)	2.0 (1.0–3.0)	0.75
Single level, No. (%)	537 (40.0)	502 (40.0)	35 (40.2)	
2–3 levels, No. (%)	671 (50.0)	627 (50.0)	44 (50.6)	
≥4 levels, No. (%)	133 (10.0)	125 (10.0)	8 (9.2)	
Operative time, median (IQR), min	195 (145–260)	195 (145–258)	205 (150–270)	0.22
Estimated blood loss, median (IQR), mL	250 (150–400)	250 (150–400)	300 (175–450)	0.08
Transfusion required, No. (%)	134 (10.0)	119 (9.5)	15 (17.2)	0.02
Perioperative nutrition care				
Preoperative dietitian consultation, No. (%)	201 (15.0)	182 (14.5)	19 (21.8)	0.07
Preoperative nutrition supplements, No. (%)	168 (12.5)	153 (12.2)	15 (17.2)	0.17
Preoperative fasting >8 h, No. (%)	503 (37.5)	471 (37.6)	32 (36.8)	0.89
Postoperative nutrition support, No. (%)	268 (20.0)	244 (19.5)	24 (27.6)	0.07
NPO duration postoperatively, median (IQR), h	12 (8–18)	12 (8–18)	14 (10–20)	0.05
First oral intake POD0-1, No. (%)	939 (70.0)	891 (71.0)	48 (55.2)	0.002

IQR, interquartile range; NPO, nil per os (nothing by mouth); PNI, Prognostic Nutritional Index; POD, postoperative day. ^a^Categories not mutually exclusive; patients may have procedures in multiple regions. Data presented as No. (%) for categorical variables and median (IQR) for continuous variables. *p*-values from Mann–Whitney U test for continuous variables and *χ*^2^ or Fisher exact test for categorical variables. Denominators explicitly stated where applicable.

### 3.4 Association between nutritional status and postoperative complications

The primary endpoint of 30-day major complications occurred in 24.0% overall (*n =* 322), with striking disparity: 40.2% in low PNI versus 22.9% in normal PNI (*p <* 0.001), corresponding to a 76% relative increase in risk (relative risk 1.76, 95% CI 1.31–2.35). Individual complications showed consistent patterns: surgical site infections (13.8% vs. 6.5%, *p =* 0.01), wound dehiscence (6.9% vs. 2.7%, *p =* 0.03), pneumonia (12.6% vs. 4.5%, *p =* 0.001), urinary tract infections (11.5% vs. 5.6%, *p =* 0.03), acute kidney injury (6.9% vs. 1.7%, *p =* 0.002), and sepsis (3.4% vs. 0.8%, *p =* 0.03).

Healthcare utilization reflected clinical impact: longer hospital stay (median 7 vs. 5 days, *p <* 0.001), with 46.0% versus 23.5% experiencing LOS > 7 days (*p <* 0.001). ICU admission rates doubled (28.7% vs. 14.0%, *p <* 0.001), with a longer duration (median 3 vs. 2 days, *p =* 0.01). Low PNI patients experienced higher rates of unplanned return to the OR (10.3% vs. 3.6%, *p =* 0.002), 30-day readmission (18.4% vs. 7.3%, *p <* 0.001), and mortality (3.4% vs. 0.3%, *p =* 0.001). An extended 90-day follow-up revealed persistent disparities in late infections, readmissions, and mortality (all *p <* 0.001; Table 2).

### 3.5 Multivariable regression analysis: independent predictors of complications

**Figure 1:**
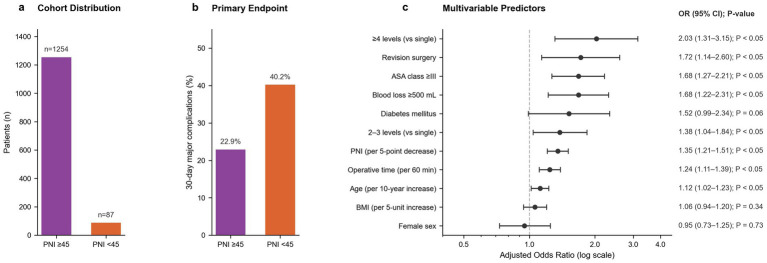
Association of prognostic nutritional index (PNI) status with postoperative outcomes. **(a)** Distribution of spine surgery patients by preoperative PNI status (normal PNI ≥ 45 vs. low PNI < 45). **(b)** Primary endpoint comparing 30-day composite complication rates between PNI groups. **(c)** Forest plot of adjusted odds ratios from multivariable logistic regression showing independent predictors of complications. Model adjusted for age, sex, BMI, ASA class, diabetes, surgical complexity, operative time, blood loss, and revision status. BMI, body mass index; ASA, American Society of Anesthesiologists; PNI, Prognostic Nutritional Index.

Multivariable logistic regression, adjusting for age, sex, BMI, ASA class, diabetes, surgical complexity, blood loss, and revision status, confirmed low PNI as an independent predictor of 30-day major complications. Model A (continuous PNI) showed that each 5-point decrease was associated with a 35% increase in the odds of complications (adjusted OR 1.35, 95% CI 1.21–1.51, *p <* 0.001). Model B (binary PNI < 45) demonstrated doubled complication odds (adjusted OR 2.02, 95% CI 1.28–3.18, *p =* 0.003). Other independent predictors included age (adjusted OR 1.12 per 10 years, *p =* 0.02), ASA class ≥III (OR 1.68, *p <* 0.001), multilevel procedures (2–3 levels: OR 1.38, *p =* 0.03; ≥4 levels: OR 2.03, *p =* 0.002), operative time (OR 1.24 per 60 min, *p <* 0.001), blood loss ≥500 mL (OR 1.68, *p =* 0.001), and revision surgery (OR 1.72, *p =* 0.01); sex and BMI were not independent predictors. Both models demonstrated good discrimination (Model A C-statistic 0.73, 95% CI 0.70–0.76; Model B 0.72, 95% CI 0.69–0.75), representing a statistically significant but numerically modest improvement over the baseline model (AUC 0.68), with acceptable calibration (Hosmer-Lemeshow *p =* 0.42 and *p =* 0.39 for Models A and B, respectively; Table 1; Figure 1C).

### 3.6 GLIM malnutrition and clinical outcomes: absence of prognostic association

GLIM-defined malnutrition, as operationalized in this study, showed no statistically significant associations with any outcome. Patients meeting GLIM criteria (*n =* 92) versus non-malnourished (*n =* 1,249) had similar rates of 30-day major complications (28.3% vs. 23.7%; unadjusted OR 1.27, 95% CI 0.79–2.03, *p =* 0.33; adjusted OR 1.18, 95% CI 0.73–1.92, *p =* 0.50). GLIM malnutrition was not associated with prolonged hospitalization (*p =* 0.32), ICU admission (*p =* 0.20), readmission (*p =* 0.79), delayed oral intake (*p =* 0.73), delayed ambulation (*p =* 0.59), or poor recovery (*p =* 0.50).

Severity grading revealed no dose–response: complication rates were 23.7% in non-malnourished, 26.5% in moderate malnutrition, and 33.3% in severe malnutrition (Cochran-Armitage trend *p =* 0.42). GLIM criteria, operationalized per consensus guidelines, lacked clinically meaningful prognostic utility in this surgical population (Table 6).

**Table 6:** GLIM malnutrition and clinical outcomes: association analysis.

Outcome	No GLIM	GLIM	Unadjusted OR (95% CI)	*p*-value	Adjusted OR (95% CI)^a^	*p*-value
	Malnutrition	Malnutrition				
	(*n =* 1,249)	(*n =* 92)				
	No. (%)	No. (%)				
30-day major complication	296 (23.7)	26 (28.3)	1.27 (0.79–2.03)	0.33	1.18 (0.73–1.92)	0.5
Hospital LOS > 7 days	308 (24.7)	27 (29.3)	1.27 (0.79–2.03)	0.32	1.21 (0.75–1.96)	0.44
ICU admission	183 (14.7)	18 (19.6)	1.42 (0.83–2.42)	0.2	1.35 (0.78–2.34)	0.29
30-day readmission	99 (7.9)	8 (8.7)	1.11 (0.52–2.36)	0.79	1.05 (0.49–2.27)	0.9
Delayed oral intake (≥POD2)	373 (29.9)	29 (31.5)	1.08 (0.69–1.71)	0.73	1.04 (0.66–1.65)	0.86
Delayed ambulation (≥POD3)	387 (31.0)	31 (33.7)	1.13 (0.72–1.77)	0.59	1.09 (0.69–1.73)	0.71
Poor recovery at 30 days^b^	249 (19.9)	21 (22.8)	1.19 (0.72–1.97)	0.5	1.14 (0.68–1.90)	0.62
GLIM malnutrition severity and outcomes (trend analysis)						
30-day major complication by GLIM severity						
No malnutrition	296/1249 (23.7%)	Reference				
Moderate malnutrition (*n =* 68)	18/68 (26.5%)	1.16 (0.66–2.03)		0.61		
Severe malnutrition (*n =* 24)	8/24 (33.3%)	1.61 (0.68–3.80)		0.28		

Cochran-Armitage test for trend: *p =* 0.42. CI, confidence interval; GLIM, Global Leadership Initiative on Malnutrition; ICU, intensive care unit; LOS, length of stay; OR, odds ratio; POD, postoperative day. ^a^Adjusted for age, sex, ASA class, number of levels, and operative time. ^b^Defined as recovery rating ≤"Fair” or pain interference ≥7/10 at 30 days. Note: GLIM diagnosis based on corrected criteria (≥1 phenotypic + ≥ 1 etiologic with CRP >5 mg/L as inflammation marker). Low prevalence (6.9%) and lack of association with outcomes suggest limited prognostic utility of GLIM in surgical populations when operationalized per consensus criteria.

### 3.7 Comparative prognostic performance: PNI superior to GLIM and constituent markers

Systematic comparison revealed marked discrimination differences. The baseline clinical model achieved an AUC of 0.68 (95% CI, 0.65–0.71). Adding continuous PNI significantly improved discrimination to an AUC of 0.73 (ΔAUC 0.05, *p <* 0.001), with a net reclassification improvement of 0.18 (95% CI 0.09–0.27), correctly reclassifying 18% of patients. Binary PNI yielded similar performance (AUC 0.72, ΔAUC 0.04, *p <* 0.001).

**Figure 2:**
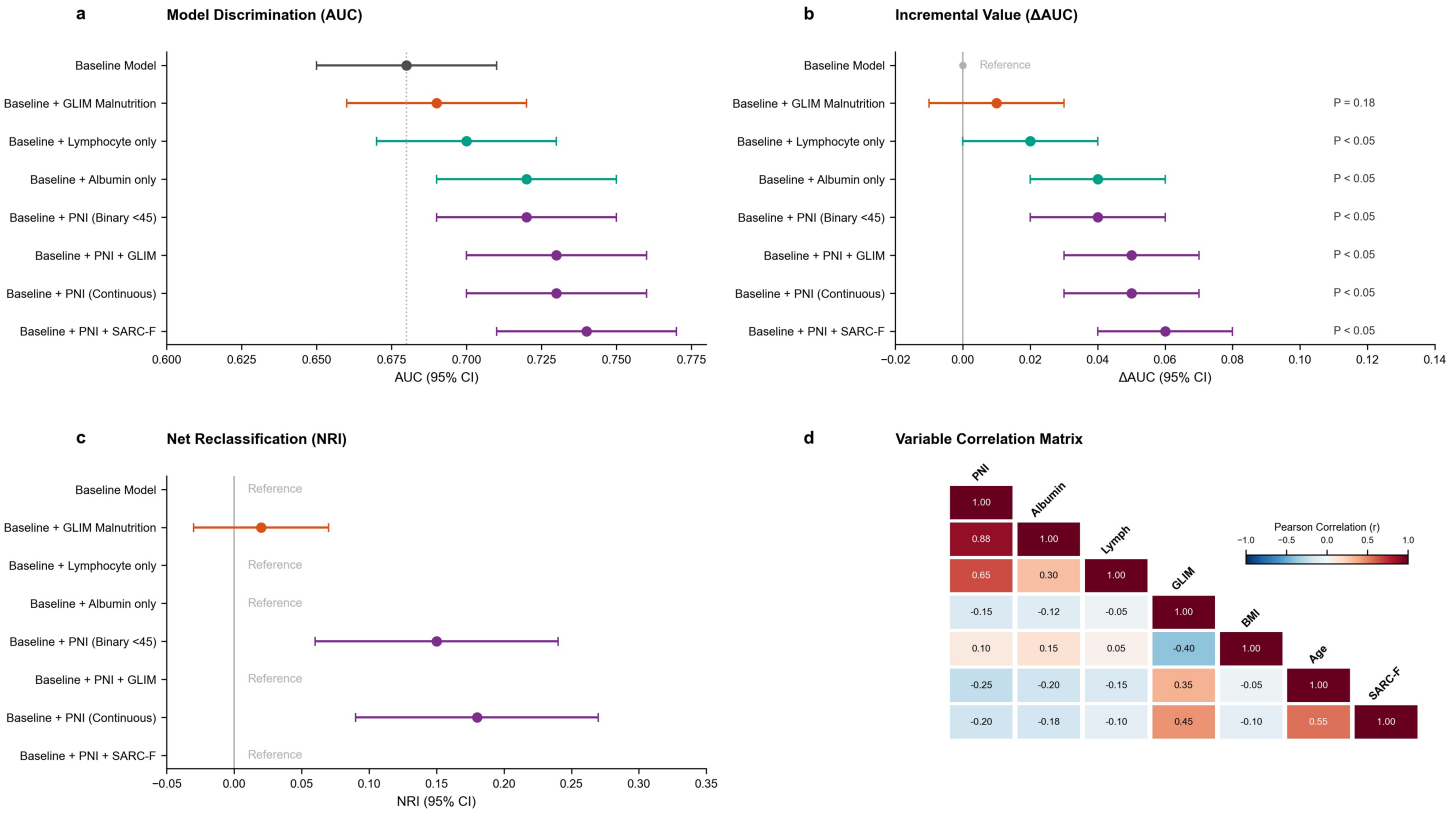
Prognostic performance and variable relationships. **(a)** Area under the receiver operating characteristic curve (AUC) comparing baseline clinical model with models augmented by nutritional markers. **(b)** Incremental discrimination improvement (ΔAUC) relative to baseline model. **(c)** Net reclassification improvement at 20% risk threshold. **(d)** Pearson correlation matrix heatmap showing relationships between nutritional markers, inflammatory parameters, and clinical variables. Color intensity indicates correlation strength (red: positive; blue: negative). AUC, area under the curve; CI, confidence interval; GLIM, Global Leadership Initiative on Malnutrition; PNI, Prognostic Nutritional Index; SARC-F, Strength, Assistance walking, Rise from chair, Climb stairs, Falls; NRI, net reclassification improvement; BMI, body mass index; CRP, C-reactive protein.

GLIM addition produced minimal improvement (AUC 0.69, ΔAUC 0.01, 95% CI − 0.01 to 0.03, *p =* 0.18) with negligible reclassification (NRI 0.02). Direct comparison confirmed PNI superiority over GLIM (ΔAUC 0.04, 95% CI 0.02–0.06, DeLong’s *p <* 0.001). When both PNI and GLIM were combined, AUC remained 0.73—identical to PNI alone—confirming GLIM added no incremental value (likelihood ratio *p =* 0.50; Figures 2a–c).

Analysis of PNI constituents showed that albumin alone achieved moderate discrimination (AUC 0.72, ΔAUC 0.04, *p <* 0.001), while lymphocyte count alone provided minimal improvement. Adding SARC-F to PNI enhanced discrimination to AUC 0.74 (ΔAUC 0.06, *p <* 0.001). Correlation analysis demonstrated strong associations with the PNI component (albumin r = 0.88, lymphocytes r = 0.65) but weak correlations with GLIM (r = −0.15) and BMI (r = 0.10), confirming distinct nutritional constructs (Table 7; Figure 2d).

**Table 7:** Prognostic nutritional index vs. GLIM malnutrition: model discrimination and incremental value.

Model	AUC (95% CI)	ΔAUC vs. Baseline (95% CI)	*p*-value^a^	Net Reclassification Improvement (95% CI)^b^
Baseline and individual nutrition predictors				
Baseline model^c^	0.68 (0.65–0.71)	Reference	–	–
Baseline + PNI (continuous)	0.73 (0.70–0.76)	0.05 (0.03–0.07)	<0.001	0.18 (0.09–0.27)
Baseline + PNI (binary <45 vs. ≥ 45)	0.72 (0.69–0.75)	0.04 (0.02–0.06)	<0.001	0.15 (0.06–0.24)
Baseline + GLIM malnutrition	0.69 (0.66–0.72)	0.01 (−0.01 to 0.03)	0.18	0.02 (−0.03 to 0.07)
Baseline + Albumin only	0.72 (0.69–0.75)	0.04 (0.02–0.06)	<0.001	–
Baseline + Lymphocyte count only	0.70 (0.67–0.73)	0.02 (0.00–0.04)	0.03	–
Combined models (Testing Incremental Value)				
Baseline + PNI + GLIM	0.73 (0.70–0.76)	0.05 (0.03–0.07)	<0.001	–
Baseline + PNI + SARC-F	0.74 (0.71–0.77)	0.06 (0.04–0.08)	<0.001	–
Direct comparison: PNI vs. GLIM				

PNI superior to GLIM: ΔAUC (PNI − GLIM) = 0.04 (95% CI, 0.02–0.06); DeLong’s test *p <* 0.001. GLIM adds no incremental value over PNI: AUC for (Baseline + PNI + GLIM) = 0.73, identical to (Baseline + PNI); likelihood ratio test *p =* 0.50. AUC, area under the receiver operating characteristic curve; CI, confidence interval; GLIM, Global Leadership Initiative on Malnutrition; PNI, Prognostic Nutritional Index; SARC-F, Strength, Assistance walking, Rise from chair, Climb stairs, Falls. ^a^*p*-value from likelihood ratio test comparing nested models. ^b^Net reclassification improvement calculated at 20% risk threshold; positive values indicate improved classification. ^c^Baseline model includes age, sex, ASA class, number of levels, operative time, blood loss ≥500 mL, and revision status.

### 3.8 Principal component analysis: multidimensional nutritional-surgical risk patterns

**Figure 3:**
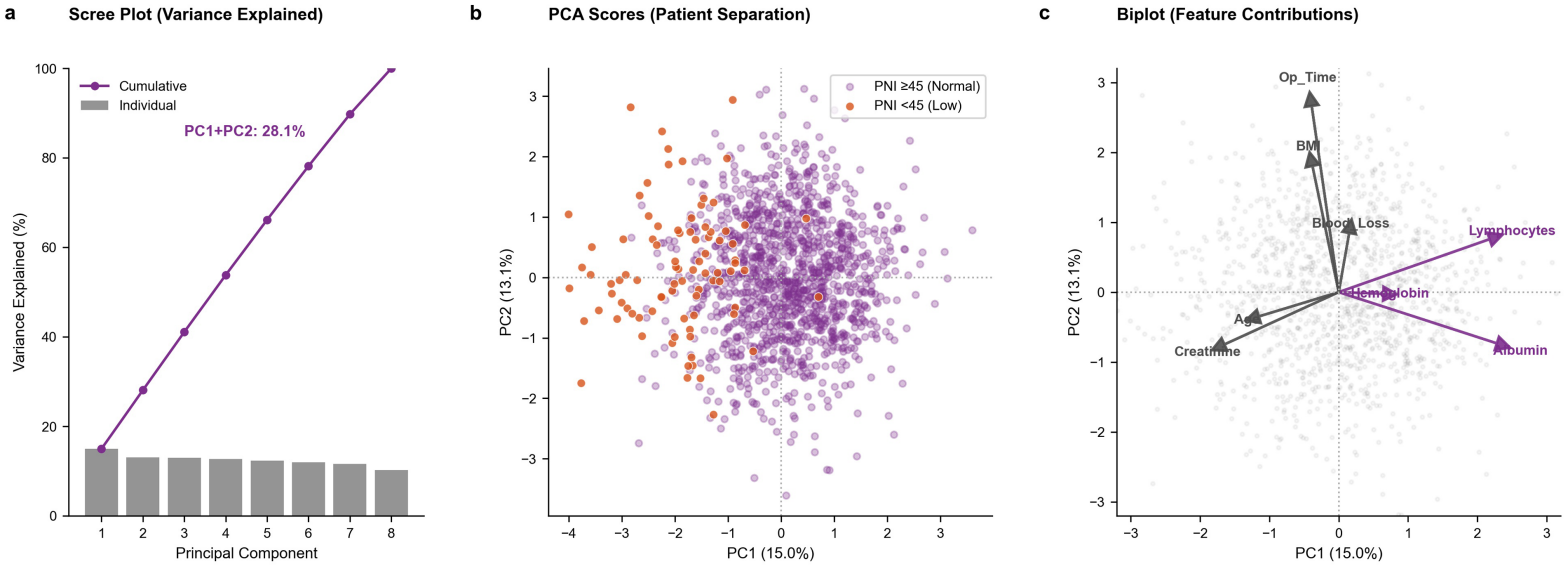
Principal component analysis (PCA) of preoperative baseline features. **(a)** Scree plot showing variance explained by individual principal components (bars) and cumulative variance (line). **(b)** Patient distribution in PC1-PC2 space stratified by PNI status, demonstrating clear separation along PC1 axis. **(c)** Biplot showing variable loadings (arrows) on PC1-PC2 axes. PC1 predominantly reflects nutritional-immune markers; PC2 captures surgical complexity and demographic factors. Analysis based on standardized values of age, BMI, operative time, blood loss, hemoglobin, albumin, lymphocyte count, and creatinine. PCA, principal component analysis; PC, principal component; PNI, Prognostic Nutritional Index; BMI, body mass index.

Principal component analysis of preoperative baseline features revealed that PC1 + PC2 explained 28.1% total variance (Figure 3a). PC1 (15.0% variance) predominantly reflected albumin, lymphocyte count, and hemoglobin levels, while PC2 (13.1% variance) captured operative complexity (operative time, blood loss) and age. Biplot visualization demonstrated clear PNI group separation along the PC1 axis, with low PNI patients clustering toward lower albumin-lymphocyte values (Figures 3b,c). This multidimensional analysis confirmed nutritional-immune status as a distinct risk dimension separable from surgical and demographic factors.

### 3.9 Longitudinal recovery trajectories: persistent disparities in patient-reported outcomes

**Figure 4:**
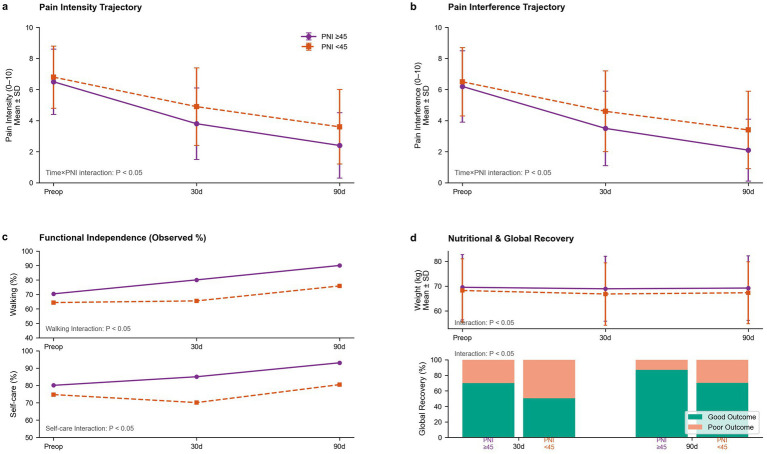
Longitudinal Recovery Trajectories by Prognostic Nutritional Index Status. **(a,b)** Mean pain intensity and pain interference scores (0–10 numeric rating scale) at preoperative, 30-day, and 90-day timepoints. **(c)** Proportion of patients achieving independent walking and self-care over time. **(d)** Top panel: mean body weight trajectories. Bottom panel: global recovery ratings at 30-day and 90-day assessments. Error bars represent standard deviation. All analyses adjusted for age, sex, number of levels, and operative time using linear mixed-effects models (continuous outcomes) or generalized estimating equations (binary outcomes). PNI, Prognostic Nutritional Index; Preop, preoperative.

Longitudinal patient-reported outcomes revealed significant persistent differences between PNI groups across 90 days. Pain intensity remained consistently higher in low PNI patients at all timepoints (preoperative 6.8 vs. 6.5, 30-day 4.9 vs. 3.8, 90-day 3.6 vs. 2.4; time × PNI interaction *p =* 0.02; Figure 4a). Pain interference showed sustained disparities (interaction *p =* 0.01; Figure 4b), indicating that compromised nutritional status was associated with slower pain resolution and functional restoration. Functional independence outcomes demonstrated striking differences in recovery patterns. By 90 days, 90.0% of patients with normal PNI achieved independent walking, compared with 75.9% of patients with low PNI (interaction *p =* 0.003). Self-care independence showed parallel disparities (93.1% vs. 80.5%, interaction *p =* 0.008). Return to work was substantially delayed: 23.9% versus 42.4% at 30 days, 62.0% versus 84.8% at 90 days (interaction *p =* 0.01; Figure 4c). Nutritional recovery metrics illustrated persistent challenges in low PNI patients: greater postoperative weight loss (14.9% vs. 7.0% with >5% loss at 30 days; time × PNI interaction *p =* 0.01), sustained appetite suppression at 90 days (75.9% vs. 89.1% normal appetite, interaction *p =* 0.05), whereas protein intake adequacy did not significantly differ (time × PNI interaction *p =* 0.18). Global recovery assessments reflected multidimensional deficits: only 50.6% rated recovery “good or better” at 30 days, increasing to 70.1% at 90 days (interaction *p =* 0.004). Surgical satisfaction showed corresponding disparities (55.2% vs. 73.1% at 30 days, 75.9% vs. 89.1% at 90 days, interaction *p =* 0.01). These exploratory findings suggest persistent differences across multiple patient-centered recovery domains in low versus normal PNI patients; given the absence of multiplicity adjustment, they are hypothesis-generating and require prospective confirmation (Table 8; Figure 4d).

**Table 8:** Patient-reported outcomes and recovery trajectories by prognostic nutritional index status.

Variable	Preoperative (baseline)	30-day postoperative	90-day postoperative	Time × PNI interaction *p*-value^a^
Pain outcomes, Mean (SD)				
Pain intensity (0–10)				0.02
PNI ≥ 45	6.5 (2.1)	3.8 (2.3)	2.4 (2.1)	
PNI < 45	6.8 (2.0)	4.9 (2.5)	3.6 (2.4)	
Pain interference with activities (0–10)				0.01
PNI ≥ 45	6.2 (2.3)	3.5 (2.4)	2.1 (2.0)	
PNI < 45	6.5 (2.2)	4.6 (2.6)	3.4 (2.5)	
Functional outcomes, No. (%)				
Independent walking				0.003
PNI ≥ 45	883 (70.4)	1,004 (80.1)	1,129 (90.0)	
PNI < 45	56 (64.4)	57 (65.5)	66 (75.9)	
Independent self-care				0.008
PNI ≥ 45	1,004 (80.1)	1,066 (85.0)	1,167 (93.1)	
PNI < 45	65 (74.7)	61 (70.1)	70 (80.5)	
Returned to work/usual activities^b^				0.01
PNI ≥ 45 (*n =* 1,036)	–	439/1036 (42.4)	879/1036 (84.8)	
PNI < 45 (*n =* 71)	–	17/71 (23.9)	44/71 (62.0)	
Nutritional recovery				
Weight (kg), mean (SD)				0.04
PNI ≥ 45	69.5 (13.2)	68.9 (13.1)	69.2 (13.0)	
PNI < 45	68.2 (12.8)	66.8 (12.6)	67.3 (12.5)	
Postoperative weight loss >5%, No. (%)				0.01
PNI ≥ 45	–	88 (7.0)	50 (4.0)	
PNI < 45	–	13 (14.9)	9 (10.3)	
Normal appetite, No. (%)				0.05
PNI ≥ 45	1,004 (80.1)	1,016 (81.0)	1,117 (89.1)	
PNI < 45	65 (74.7)	61 (70.1)	66 (75.9)	
Protein ≥2 servings/day, No. (%)				0.18
PNI ≥ 45	940 (75.0)	978 (78.0)	1,029 (82.1)	
PNI < 45	62 (71.3)	61 (70.1)	66 (75.9)	
Global recovery assessment, No. (%)				
Recovery rating “Good” or better				0.004
PNI ≥ 45	–	879 (70.1)	1,092 (87.1)	
PNI < 45	–	44 (50.6)	61 (70.1)	
Satisfied with surgical outcome				0.01
PNI ≥ 45	–	916 (73.1)	1,117 (89.1)	
PNI < 45	–	48 (55.2)	66 (75.9)	

PNI, Prognostic Nutritional Index. ^a^From linear mixed-effects models (continuous outcomes) or generalized estimating equations with logit link (binary outcomes) testing whether trajectory over time differs by PNI status. Models adjusted for age, sex, and surgical complexity (number of levels, operative time). Missing data handled by maximum likelihood (mixed models) or available case analysis (GEE). Time windows: preoperative (within 7 days before surgery), 30-day (25–35 days postoperative), 90-day (80–100 days postoperative). ^b^Denominator restricted to those employed or in usual role at baseline (*n =* 1,107 overall; *n =* 1,036 PNI ≥45; *n =* 71 PNI <45). Explicitly stated to ensure clarity. Data presented as mean (SD) for continuous variables and No. (%) for categorical variables. Significant time × PNI interaction indicates that recovery trajectory differs between PNI groups (*p <* 0.05). Low PNI patients show persistently slower improvement across multiple recovery domains.

## 4 Discussion

This cohort of 1,341 spinal surgery patients reflects an important divergence in the approach to nutritional assessment: preoperative PNI < 45 is an independent predictor of major complications, 76% increase of risk (40.2 vs. 22.9%, *p <* 0.001), whereas malnutrition defined by the pragmatic GLIM operationalization showed no statistically significant prognostic association with any outcome in this cohort. The near-complete absence of positive agreement between these tools (Cohen’s *κ* = 0.00; positive agreement 5.6%, negative agreement 93.2%; McNemar *χ*^2^ = 0.15, *p =* 0.70), with 94.3% of biochemically high-risk PNI patients classified as GLIM-negative, raises important questions about the construct validity of malnutrition diagnosis in elective spine surgery populations. These findings suggest that PNI offers a practical, readily available complement to formal nutritional frameworks for perioperative risk stratification; however, they do not establish that the GLIM framework itself lacks surgical validity, as our operationalization almost certainly underestimates GLIM’s sensitivity relative to implementations using CT- or DXA-based muscle assessment and prospectively collected phenotypic data.

Our findings corroborate accumulating evidence for PNI’s predictive capacity. A 2024 meta-analysis of 10 spine surgery studies (*n =* 3,249) reported that low PNI increased overall complications by 1.82-fold (95% CI, 1.42–2.34), surgical site infections by 1.41-fold, and delirium by 2.36-fold (6). Oe et al. found 49% versus 23% complication rates (*p <* 0.001) in adult spinal deformity patients, stratified by PNI < 50 (46), while Ushirozako et al. (38) demonstrated a 6% increase in infection risk per unit decrease in PNI. Our observed adjusted OR of 2.02 closely approximates the meta-analytic pooled estimate of 1.82 (95% CI 1.42–2.34), providing external validation and supporting generalizability beyond single-center case-mix. In addition, the evidence for the superiority of PNI in continuous (OR 1.35 per 5-point decrease) and binary (OR 2.02 for <45) formulations provides both evidence of dose–response relationships and clinical decisiveness. Regarding the choice of threshold, while PNI < 40 represents the original Buzby cutoff for severe immunonutritional depletion in gastrointestinal surgery, this conservative boundary identifies only profound deficiency and would classify only 14 patients (1.0%) in our cohort—a prevalence too low to power meaningful clinical risk stratification. In elective orthopedic spine populations, where patients undergo careful preoperative selection and frank nutritional deficiency is less prevalent than in GI oncology, the PNI < 45 threshold captures a broader but clinically relevant intermediate-risk group, consistent with its adoption in the spine surgery literature (38, 76) and with our ROC-derived Youden index optimization (38). The continuous PNI model (OR 1.35 per 5-point decrease) further confirms a monotonic dose–response relationship, supporting the biological plausibility of risk gradients across the PNI spectrum below 45. Second, our 90-day longitudinal outcomes demonstrate that PNI predicts not only complications but also functional recovery, pain resolution, return to work, and patient satisfaction—domains that are comprehensive and patient-centered and are rarely examined in surgical nutrition research. Third, a direct head-to-head comparison with GLIM establishes PNI’s incremental value (ΔAUC 0.04, *p <* 0.001) and GLIM’s null contribution when combined with PNI (AUC unchanged at 0.73, likelihood ratio *p =* 0.50).

The critical role of albumin—PNI’s dominant component—is established in numerous spine surgery studies examining hypoalbuminemia independently. A recent systematic review and meta-analysis of degenerative and deformity correcting spine operations corroborated the finding that hypoalbuminemia is associated with significantly increased postoperative complications, although findings among studies differed based on the surgical populations and albuminemia thresholds (46). Chaker et al. (47), using a cohort of 22,518 patients from the Michigan Spine Surgery Improvement Collaborative registry, showed that even borderline albumin levels (3.5–4.0 g/dL) were important for 30-day and 90-day readmission rates and increased length of hospitalization in lumbar surgery. This dose-dependent relationship mirrors our continuous PNI findings, where each 5-point decrease escalated complication odds 35%.

In osteoporotic vertebral compression fractures, severe hypoalbuminemia (<2.5 g/dL) was associated with mortality OR 2.96 and major complications OR 2.48 compared with major complications compared to normal albumin (3.5 g/dL) in some studies (48, 49). Similarly, Gelfand et al. (50) reported 44.4% 30-day mortality in severely hypo-albuminemic patients undergoing metastatic spine surgery versus 7.6% in normo-albuminemic patients—a sevenfold disparity underscoring albumin’s prognostic potency in high-risk populations. Analysis of 627 patients with primary pyogenic spinal infections by Camino-Willhuber et al. identified hypoalbuminemia as the most significant risk factor for unplanned readmission and reoperation, with dialysis as the second (51). Our PNI < 45 patients had a 17.2% transfusion requirement compared to 9.5% in normal PNI patients, similar to Hussain et al., who demonstrated that hypoalbuminemia was associated with a 1.4-fold increased risk of transfusion in spinal metastasis decompression, with 43% transfusion rates in hypo-albuminemic patients during the perioperative period (52).

Critically, several studies documenting albumin’s null associations with specific outcomes paradoxically support PNI’s superiority over albumin alone. A study found no association between hypoalbuminemia and surgical site infections following spinal fusion (*n =* 602), but since PNI also incorporates lymphocyte-mediated immune surveillance, albumin alone was a significant predictor in their cohort (53). This mechanistic complementarity explains why PNI (AUC 0.73) outperformed albumin alone (AUC 0.72) in our discriminative analyses, despite albumin’s high correlation with PNI (r = 0.88). The lymphocyte component, though with a lower individual AUC (0.70), contained non-redundant immunologic information that captures cell-mediated immunity, fundamental to wound healing and resistance to infection.

GLIM’s lack of predictive power, despite the 6.9% prevalence of malnutrition (similar to our low PNI prevalence of 6.5%), requires a mechanistic explanation. In our cohort of 87 low PNI patients who demonstrated a 76% higher risk of complications, only 5 (5.7%) qualified for GLIM, indicating 94.3% discordance and suggesting that GLIM’s phenotypic requirements (weight loss, low BMI, muscle wasting) fail to detect biochemically evident immune-nutritional compromise. Conversely, 87 normal PNI patients meeting GLIM criteria showed no excess surgical risk, indicating that GLIM identifies phenotypic changes that were not associated with excess surgical risk in this acute perioperative settings. GLIM’s requirement for simultaneous phenotypic and etiologic criteria creates restrictive thresholds. While the prevalence of inflammation (CRP > 5 mg/L) was 66.0%, this ubiquity in degenerative spine disease undermined discriminative capacity. When the inflammatory criterion is met in two-thirds of patients, the diagnosis of malnutrition is rate-limited by the phenotypic criteria, which is precisely where GLIM shows poor sensitivity in surgical spine populations. The differential validity of GLIM across disease domains reflects fundamental differences in the pathophysiology of malnutrition. In gastrointestinal diseases, including GI malignancies, inflammatory bowel disease, and post-resection states, malnutrition arises from malabsorption, tumor-related catabolism, and impaired enteral intake, which reliably produce the phenotypic changes—progressive weight loss, muscle wasting, and low BMI—that GLIM’s phenotypic criteria are specifically constructed to detect. Validation studies in GI oncology and GI surgery have accordingly demonstrated strong associations with GLIM outcomes because the phenotypic signal is robust and clinically conspicuous. In contrast, degenerative spinal disease is characterized by chronic mechanical dysfunction and low-grade neuroinflammation rather than systemic catabolic disease; patients frequently maintain body weight and anthropometric bulk while harboring occult biochemical immune-nutritional compromise. The result is a phenotypic-etiologic mismatch: GLIM’s etiologic inflammation criterion is almost universally satisfied in this population (CRP > 5 mg/L in 66.0% of patients), yet phenotypic thresholds remain largely unmet (31.6% any phenotypic criterion), creating a diagnostic bottleneck that excluded 94.3% of biochemically high-risk PNI patients from GLIM classification. Moreover, perioperative risk in spine surgery is governed by current functional immune competence—captured by albumin-mediated immune modulation and lymphocyte-mediated wound surveillance—rather than by cumulative documentation of phenotypic depletion over preceding months, which is the process GLIM was architecturally designed to characterize. This mechanistic mismatch explains why GLIM, though validated in GI disease, demonstrated no prognostic utility in our spine surgery cohort, and underscores the importance of population-specific validation before cross-domain extrapolation of nutritional assessment frameworks. Zhou et al. (54) found that PNI detected 60.24% malnutrition, compared with 36.75% according to GLIM, in hepatobiliary surgery, with weak concordance (k = 0.265). Validation studies report GLIM sensitivities ranging from 20 to 93.7%, depending on muscle mass assessment methods (54–56), reflecting fundamental measurement heterogeneity. Emergency gastrointestinal surgery studies showed GLIM predicted outcomes only when albumin was added as a phenotypic criterion (57)—essentially reconstituting PNI within GLIM’s framework.

The broader GLIM validation literature reveals systematic implementation challenges explaining our null findings. Jazinaki et al. (20) prospective validation showed their test GLIM had a dramatic range in its accuracy depending on the measurement of muscle mass used in the validation: calf circumference versus mid-upper arm circumference gave different sensitivities and specificities, with some methods showing no association with mortality. This measurement-dependent validity has a direct effect on surgical applications in which gold standard imaging (DXA, CT, MRI) is rarely feasible preoperatively. We used calf circumference according to Asian Working Group criteria; however, only 13.9% met the reduced muscle mass threshold, despite 66% meeting the inflammatory criteria, resulting in a diagnostic bottleneck that excluded 94.3% of high-risk PNI patients from GLIM classification.

Recent GLIM consensus updates acknowledge these challenges. Jensen et al. (34) 2024 five-year review, synthesizing >400 publications, emphasized that “assessment of low muscle mass should be guided by experience and available technological resources” and “clinical judgment may suffice” for inflammation. This flexibility, designed for global applicability, paradoxically introduces heterogeneity undermining surgical validity. The 2023 GLIM inflammation guidance recognized that surgical patients with major surgery or trauma “may not initially meet phenotypic GLIM criteria, but readily meet the etiologic criterion” and “should be assumed at elevated risk” even without a formal malnutrition diagnosis (58)—effectively conceding GLIM’s phenotypic criteria fail to capture acute surgical risk.

Tan et al.’s validation in 1,115 cancer patients undergoing major abdominal surgery found GLIM predicted infection-related (OR 2.19) and wound-healing complications (OR 2.54) when implemented with screening, structured assessment, and CT-based muscle evaluation (22). However, our pragmatic implementation—lacking routine preoperative CT, prospective documentation of weight loss, and formal dietary assessment—reflects the capabilities typical of surgical centers. Murnane et al. (59) demonstrated that the CT-based skeletal muscle index for GLIM identified 76.6% of patients with malnutrition in esophagogastric cancer surgery and predicted complications, illustrating technology-dependent performance. GLIM’s surgical validity requires resource-intensive assessments that are unavailable in most perioperative settings, whereas PNI relies on routine laboratory values, ensuring universal applicability.

Modified GLIM approaches further illustrate this limitation. Haines et al. added albumin ≤3.5 g/dL as a phenotypic criterion (integrating half of PNI) and designated emergency surgery as an etiologic inflammatory criterion, finding that this modified framework predicted adverse outcomes (57). This modification’s success, paradoxically, validates our findings: GLIM works when reconstituted to include PNI components but fails when implemented per the original specifications.

PNI’s prognostic capacity derives from capturing functional immune-nutritional reserves rather than documenting phenotypic depletion. Albumin modulates immunity through prostaglandin E2 sequestration (60), neutrophil antimicrobial enhancement (61), B-cell expansion (62), and upregulation of NF-κB-mediated tissue repair (63). Hypoalbuminemia predicts surgical site infections across specialties (64, 65) and immune checkpoint inhibitor efficacy in oncology (66, 77), serving as an integrative immune-competence biomarker. Lymphocytes represent cell-mediated immunity, which is fundamental to wound healing and the stress response (67). PNI’s formula (10 × albumin + 0.005 × lymphocytes) creates a weighted composite that favors albumin while incorporating immune capacity—empirically validated over decades in surgery (68, 69). GLIM documents cumulative phenotypic changes (muscle loss over months) while PNI captures current functional capacity. Surgical stress demands immediate immune competence, not gradual depletion of documentation.

PNI integration into routine preoperative assessment for spine surgery is immediately feasible and requires only standard laboratory values. The 40.2% complication rate, higher ICU admission rates (28.7% vs. 14.0%), extended hospitalization (median 7 vs. 5 days), and higher 30-day mortality (3.4% vs. 0.3%) in low PNI patients represent a substantial risk burden associated with preoperative immune-nutritional compromise; whether this risk is directly modifiable through nutritional prehabilitation requires evidence from randomized controlled trials. However, low PNI patients received only marginally higher nutritional interventions (dietitian consultation: 21.8% vs. 14.5%, *p =* 0.07), indicating practice gaps that PNI-based screening protocols could help address. Future studies are needed to determine whether targeted interventions (protein augmentation, immunological-enhancing formulas, preoperative optimization) can improve outcomes in low PNI candidates (68–70). Our 90-day recovery data highlight that PNI predicts comprehensive patient-focused outcomes such as functional independence, return to work, and surgical satisfaction, which aligns with modern value-based care metrics that focus on patient-reported outcomes beyond traditional complications. This temporal persistence suggests that PNI captures a fundamental physiological reserve associated with both acute stress response and sustained recovery capacity.

These findings should not be taken as a wholesale rejection of GLIM, but rather as highlighting its context-dependent validity. GLIM may effectively identify chronic malnutrition in medical populations or cancer cachexia, where phenotypic changes predominate, but lacks discriminative capacity when functional immune-nutritional status determines perioperative risk. GLIM documents cumulative depletion (weight loss over months, muscle wasting) while surgical stress demands current immune competence. Future GLIM revisions should consider incorporating albumin/lymphocyte parameters as phenotypic criteria, relaxing simultaneous phenotypic/etiologic requirements in surgical contexts, or developing surgery-specific inflammatory thresholds beyond crude CRP > 5 mg/L cutpoints. Parallel frameworks may be necessary—GLIM for chronic malnutrition characterization, PNI for acute surgical risk stratification. Emerging evidence suggests that combined tools may be synergistic; our data showing that PNI + SARC-F improved AUC from 0.73 to 0.74 support this approach (70, 71).

Strengths of our study include prospective design, consecutive enrollment to minimize selection bias, large cohort, allowing robust multivariable adjustment including surgical complexity parameters, seldom controlled in nutritional studies, head to head PNI-GLIM comparison using identical populations to eliminate confounding by indication, 90-day longitudinal patient-reported outcomes that extends beyond traditional complications, rigorous statistical validation (net reclassification improvement, calibration assessment, principal component analysis demonstrating PNI as separate risk dimension). A pragmatic single-cohort, center-based design improves generalizability to community practice outside academic centers.

Several important limitations warrant careful interpretation. First, the single-center design at a Chinese tertiary academic center limits generalizability. The lower malnutrition prevalence (6.5–6.9%) compared with Western spine cohorts (15–30%) may reflect tertiary referral selection bias, distinct nutritional epidemiology, or cultural dietary patterns. External validation across diverse healthcare settings, geographic regions, and higher-risk populations is essential. Second, GLIM operationalization using pragmatic measures (calf circumference and CRP only) rather than imaging-based assessments (CT, DXA), exclusion of ASA classification, and retrospective documentation of weight loss likely reduced sensitivity compared to optimal implementation. These methodological decisions reflect real-world constraints but preclude definitive conclusions about GLIM’s inherent capacity relative to our implementation. Comprehensive GLIM studies with prospective monitoring and imaging-based assessment of sarcopenia may demonstrate stronger associations. Third, low event rates limit precision—only 87 low PNI patients (6.5%), rare complications (sepsis *n =* 13, CSF leak *n =* 27), and mortality (30-day *n =* 7, 90-day *n =* 13) produce wide confidence intervals and preclude adequately powered subgroup analyses. Multiple secondary outcomes tested without multiplicity adjustment may inflate the type I error rate, though the prespecified composite primary endpoint mitigates false-positive findings. Additionally, although the PNI < 45 threshold was prespecified from external literature, the post-hoc confirmatory ROC analysis on the current dataset introduces partial circularity when interpreting the AUC for Model B; the binary-model AUC of 0.72 reflects threshold-based classification using a cutoff confirmed rather than derived on this cohort, and formal optimism correction for cutoff selection was not performed. Fourth, observational design precludes causal inference despite multivariable adjustment. Three pre-specified analyses were not computable from the current dataset and represent important limitations. First, a strictly preoperative multivariable model excluding intraoperative covariates (operative time, estimated blood loss) was not derived; the reported Models A and B therefore include variables unavailable at the moment of preoperative decision-making, which limits the precision of the preoperative risk-stratification claim. Second, extended calibration metrics including Brier score, calibration intercept and slope, bootstrap internal validation, and decision-curve analysis were not performed; model performance is therefore characterized by the C-statistic (0.73 and 0.72 for Models A and B) and Hosmer-Lemeshow test (*p =* 0.42 and *p =* 0.39) only, which limits conclusions about absolute calibration accuracy and net clinical benefit at threshold probabilities. Third, pre-specified sensitivity analyses sequentially adjusting for preoperative CRP, active malignancy, urgency of surgery, baseline walking-aid use, baseline pain interference, and SARC-*F* ≥ 4 as a surrogate frailty indicator were not computable from the available aggregated data; whether the PNI–complication association is attenuated by these correlated determinants of surgical risk therefore remains formally undemonstrated. Albumin is an acute-phase reactant that falls with inflammation independently of nutritional depletion; low PNI may therefore partly reflect inflammatory burden or disease severity rather than a modifiable nutritional state. Causal attribution requires randomized prehabilitation trials. Unmeasured confounding by cognitive impairment, socioeconomic factors, or health behaviors (smoking, alcohol) also remains possible. Low PNI may mark underlying frailty rather than represent an independent causal pathway. Fifth, missing longitudinal data (4.0% at 30 days, 6.6% at 90 days), though low, may introduce bias if non-random. Patients with poor outcomes may differentially withdraw. Return-to-work analyses excluded non-employed patients, limiting generalizability. Sixth, the absence of PNI-guided interventions prevents assessment of whether optimization improves outcomes. Our data demonstrate prognostic capacity but cannot establish whether targeting low PNI patients with protein supplementation, immune-enhancing nutrition, or delayed surgery would modify risk, a question that requires randomized trials. Marginally higher interventions in low PNI patients (dietitian consultation 21.8% vs. 14.5%, *p =* 0.07) were non-significant and reflect clinical judgment rather than protocol-driven care. Seventh, temporal changes over 3.5 years and non-uniform ERAS implementation may introduce heterogeneity affecting PNI calibration. Finally, predominantly elective degenerative surgery (84.9, 75.0%) with limited emergency procedures (15.1%), trauma (12.5%), or oncologic cases (5.0%) restricts generalizability. PNI’s performance may differ in emergency trauma, cancer cachexia, or complex reconstructions. Ethnic homogeneity limits applicability to diverse populations with different nutritional patterns, body composition norms, and albumin reference ranges.

## 5 Conclusion

This prospective cohort demonstrates that preoperative PNI independently predicts postoperative complications, healthcare utilization, and 90-day recovery trajectories in spine surgery. PNI demonstrated greater discriminative performance than the pragmatic GLIM operationalization used here, which relied on calf circumference for muscle mass assessment, CRP for inflammation, and retrospectively collected weight-loss data; this finding should not be interpreted as establishing the superiority of PNI over the GLIM framework when implemented with optimal imaging-based phenotypic assessment. However, GLIM implementation limitations (anthropometric rather than imaging-based assessment, ASA exclusion, and retrospective phenotypic data) prevent definitive conclusions about the framework’s inherent capacity relative to our specific operationalization. PNI assessment requires only routine preoperative laboratory values, making it simple and cost-effective. High-risk patients (PNI < 45) experiencing 76% increased complications, doubled ICU admissions, and impaired functional recovery represent intervention targets. However, whether PNI-guided optimization—protein supplementation, immune-enhancing nutrition, or delayed surgery—actually improves outcomes remains to be shown by prospective randomized trials. Future priorities include: multicenter external validation across diverse settings and populations; interventional trials that randomize low PNI patients to intensive prehabilitation versus standard care; mechanistic studies that elucidate albumin-lymphocyte pathways; hybrid assessment frameworks that integrate PNI with comprehensive phenotypic evaluation; and health economic analyses of PNI-based screening cost-effectiveness. PNI emerges as pragmatic and clinically informative for preoperative risk stratification. At the same time, GLIM’s acute surgical role requires clarification through studies that optimize implementation, use imaging-based assessment, and collect prospective data. Importantly, prospective interventional trials are essential for determining whether PNI-guided optimization strategies can improve patient outcomes.

## Data Availability

The raw data supporting the conclusions of this article will be made available by the authors, without undue reservation.
