# Supplementary material for: Prognostic nutritional index versus pragmatically operationalized GLIM criteria for predicting postoperative complications and recovery in spine surgery: a prospective cohort study
Source: Front Nutr. 2026 Jun 19;13:1767984. doi: 10.3389/fnut.2026.1767984 (PMC13327899; doi:10.3389/fnut.2026.1767984)
Supplement: Supplementary file 1 [file Table_1.DOCX]

**STROBE Statement — Checklist of items that should be included in reports of observational studies**

**Study type: Cohort**

**Manuscript title:** Prognostic Nutritional Index versus Pragmatically Operationalized GLIM Criteria for Predicting Postoperative Complications and Recovery in Spine Surgery: A Prospective Cohort Study

**Authors:** YongBo Yang, Rui Shi, Jian Zhou, Zhenjun Zhu

**Reference:** *von Elm E, Altman DG, Egger M, Pocock SJ, Gøtzsche PC, Vandenbroucke JP; STROBE Initiative. The Strengthening the Reporting of Observational Studies in Epidemiology (STROBE) statement: guidelines for reporting observational studies. Lancet. 2007;370(9596):1453–1457.*

| **Item No.** | **Recommendation** | **Location in Manuscript** | **Reported?** |
| --- | --- | --- | --- |
| **TITLE AND ABSTRACT** | | | |
| 1a | Indicate the study's design with a commonly used term in the title or the abstract. | Title: "A Prospective Cohort Study"; Abstract Methods: "Prospective outcome cohort with retrospectively abstracted baseline data" | Yes |
| 1b | Provide in the abstract an informative and balanced summary of what was done and what was found. | Abstract — Background, Methods, Results, Conclusion; primary endpoint, PNI vs GLIM discrimination, and exploratory secondary findings reported; condensed per reviewer comment | Yes |
| **INTRODUCTION** | | | |
| 2 | Explain the scientific background and rationale for the investigation being reported. | Introduction §1 — global burden of spinal pathology; malnutrition-outcomes link; limitations of BMI; rationale for PNI-GLIM head-to-head comparison in Chinese spine surgery cohort | Yes |
| 3 | State specific objectives, including any prespecified hypotheses. | Introduction §1 final paragraph — to compare predictive value of PNI versus GLIM on 30-day complications, hospital length of stay, and 90-day functional recovery trajectories | Yes |
| **METHODS** | | | |
| 4 | Present key elements of study design early in the paper. | Methods §2.1 — "prospective outcome cohort study with retrospectively abstracted baseline data"; single-centre; February 2022–September 2025 | Yes |
| 5 | Describe the setting, locations, and relevant dates, including periods of recruitment, exposure, follow-up, and data collection. | Methods §2.1 — Xinxiang Central Hospital, The Fourth Clinical College of Henan Medical University, Henan, China; recruitment February 2022–September 2025; 30-day and 90-day follow-up windows stated | Yes |
| 6a | Give the eligibility criteria, and the sources and methods of selection of participants. Describe methods of follow-up. | Methods §2.1 — consecutive adults ≥18 y; elective/urgent cervical/thoracic/lumbar spine surgery; complete preoperative albumin and lymphocyte count within 7 days; detailed inclusion and exclusion criteria listed; mandatory 30-day follow-up with prospective outcome assessment | Yes |
| 6b | For matched studies, give matching criteria and number of exposed and unexposed. | Not applicable — unmatched prospective cohort design | N/A |
| 7 | Clearly define all outcomes, exposures, predictors, potential confounders, and effect modifiers. Give diagnostic criteria, if applicable. | Methods §2.2 (PNI formula; GLIM phenotypic/etiologic criteria with thresholds); §2.3 (demographics, comorbidities); §2.4 (surgical variables); §2.6.1 (composite outcome with Clavien-Dindo ≥II anchor; CDC/NHSN SSI; KDIGO AKI; Sepsis criteria); §2.6.3 (PRO instruments) | Yes |
| 8* | For each variable of interest, give sources of data and details of methods of assessment (measurement). Describe comparability of assessment methods if there is more than one group. | Methods §2.2.1 (albumin by bromocresol green; lymphocytes by automated CBC within 7 days preoperatively); §2.2.2 (calf circumference measured at 90° knee flexion; CRP by high-sensitivity immunoturbidimetric assay; structured dietary recall by trained nutritionists); §2.6.3 (PRO assessment windows: 25–35 days, 80–100 days) | Yes |
| 9 | Describe any efforts to address potential sources of bias. | Methods §2.1 (consecutive enrolment to minimise selection bias; blinded PRO coordinators; standardised perioperative protocols); §2.7 (multivariable logistic regression with 10 prespecified confounders; EPV = 29.3); Limitations §4 (residual confounding, differential withdrawal, temporal heterogeneity) | Yes |
| 10 | Explain how the study size was arrived at. | Methods §2.1 — all consecutive eligible patients enrolled over the full study period (n=1,341); no formal a priori sample size calculation performed; study was hypothesis-generating with consecutive enrolment. Acknowledged in Limitations §4 (paragraph 3) | Partial |
| 11 | Explain how quantitative variables were handled in the analyses. If applicable, describe which groupings were chosen and why. | Methods §2.2.1 — PNI modelled as continuous (per 5-point decrease; Model A) and binary (<45 vs ≥45; Model B); threshold prespecified from Ushirozako et al. 2021 and Oe et al. 2020 external literature; post-hoc confirmatory ROC/Youden index analysis on current cohort reported; §2.7 — blood loss dichotomised at ≥500 mL | Yes |
| 12a | Describe all statistical methods, including those used to control for confounding. | Methods §2.7 — Mann-Whitney U test; chi-square/Fisher exact; multivariable logistic regression (Models A and B, 11 parameters each); Hosmer-Lemeshow calibration; DeLong AUC comparison; NRI at 20% threshold (justified); Cochran-Armitage trend test; linear mixed-effects models; GEE with logit link | Yes |
| 12b | Describe any methods used to examine subgroups and interactions. | Methods §2.7 — subgroup analyses by age (<65/≥65 years), surgical approach (posterior/anterior), primary indication (degenerative/other); time × PNI interaction terms in mixed-effects models and GEE for longitudinal PRO analyses | Yes |
| 12c | Explain how missing data were addressed. | Methods §2.7 — maximum likelihood estimation for mixed-effects models; available case analysis for GEE; missingness patterns compared between PNI groups (p=0.32 at 30 days; p=0.28 at 90 days by chi-square); §2.6.3 — follow-up completion rates reported (96.0% at 30d; 93.4% at 90d) | Yes |
| 12d | If applicable, explain how loss to follow-up was addressed. | Methods §2.6.3 — 1,287/1,341 (96.0%) retained at 30 days; 1,253/1,341 (93.4%) at 90 days; missingness non-differential between PNI groups; Limitations §4 paragraph 5 — potential for differential withdrawal acknowledged | Yes |
| 12e | Describe any sensitivity analyses. | Methods §2.7 — four prespecified sensitivity analyses: (1) excluding emergency surgery; (2) stratification by surgical complexity; (3) alternative PNI cutoffs (quartile-based); (4) sequential confounder adjustment for CRP, malignancy, urgency, baseline function, SARC-F ≥4. Note: analysis (4) not computable from available aggregated data — transparently acknowledged in Limitations §4 paragraph 4 | Partial |
| **RESULTS** | | | |
| 13a | Report numbers of individuals at each stage of study — e.g. numbers potentially eligible, examined for eligibility, confirmed eligible, included in the study, completing follow-up, and analysed. | Results §3.1 — 1,341 consecutive eligible patients enrolled; 1,287 (96.0%) at 30 days; 1,253 (93.4%) at 90 days; exclusion criteria listed in Methods §2.1 | Partial |
| 13b | Give reasons for non-participation at each stage. | Methods §2.1 — exclusion criteria enumerated by category; individual exclusion counts per criterion not reported; no participant flow diagram included. Acknowledged in Limitations §4 | Partial |
| 13c | Consider use of a flow diagram. | No participant flow diagram included in this version | No |
| 14a | Give characteristics of study participants and information on exposures and potential confounders. | Results §3.1; Table 1 — full baseline characteristics by PNI group: demographics, comorbidities (ASA class, DM, CKD, CVD, malignancy), preoperative laboratory values (albumin, lymphocytes, Hb, CRP, creatinine), baseline functional and pain status | Yes |
| 14b | Indicate number of participants with missing data for each variable of interest. | Methods §2.6.3 — overall missingness reported (4.0% at 30d; 6.6% at 90d) and shown to be non-differential; individual variable-level missing counts not reported | Partial |
| 14c | Summarise follow-up time (e.g. average and total amount). | Methods §2.6.3 — prespecified follow-up windows: 30-day (25–35-day window), 90-day (80–100-day window); retention rates reported (96.0% and 93.4%) | Yes |
| 15 | Report numbers of outcome events or summary measures over time. | Results §3.4; Table 4 — 30-day composite events (n=322, 24.0% overall; 40.2% vs 22.9% by PNI group); individual complication counts; ICU admission, LOS, readmission, mortality; 90-day extended outcomes; Table 8 — longitudinal PRO values at three timepoints | Yes |
| 16a | Give unadjusted and, if applicable, adjusted estimates and their precision (e.g., 95% CI). Make clear which confounders were adjusted for and why they were included. | Results §3.5; Table 5 — unadjusted and adjusted ORs with 95% CIs for both Models A (continuous PNI) and B (binary PNI); 10 covariates selected a priori (age, sex, BMI, ASA class, DM, vertebral levels, operative time, blood loss, revision status); EPV = 29.3; Table 6A — GLIM unadjusted and adjusted ORs | Yes |
| 16b | Report category boundaries when continuous variables were categorised. | Methods §2.2.1 — PNI <45 vs ≥45 (prespecified from literature); §2.2.2 — GLIM BMI thresholds (<20 if <70 y; <22 if ≥70 y); blood loss ≥500 mL; calf circumference <34 cm (men), <33 cm (women) | Yes |
| 16c | If relevant, consider translating estimates of relative risk into absolute risk for a meaningful time period. | Results §3.4 — absolute complication rates reported (40.2% vs 22.9%); relative risk 1.76 (95% CI 1.31–2.35) reported alongside adjusted OR | Yes |
| 17 | Report other analyses done — e.g. analyses of subgroups and interactions, and sensitivity analyses. | Results §3.2 (PNI-GLIM concordance with PA/NA/McNemar); §3.6 (GLIM severity dose-response; Cochran-Armitage p=0.42); §3.7 (AUC comparison across 8 models; NRI); §3.8 (PCA); §3.9 (longitudinal mixed-effects and GEE with time × PNI interaction); Sensitivity analysis (4) results not reported — see Limitations §4 | Partial |
| **DISCUSSION** | | | |
| 18 | Summarise key results with reference to study objectives. | Discussion §4 opening paragraph — PNI independently predicts 30-day complications (OR 2.02); GLIM null (OR 1.18); near-complete absence of positive agreement (κ=0.00); key findings contextualised against 2024 meta-analytic estimate (OR 1.82) | Yes |
| 19 | Discuss limitations of the study, taking into account sources of possible bias or imprecision. Discuss both direction and magnitude of any potential bias. | Limitations §4 — eight limitations: (1) single-centre generalisability; (2) pragmatic GLIM operationalisation; (3) low event rates and wide CIs; (4) three unperformed pre-specified analyses (Model C, calibration metrics, sensitivity analysis 4); (5) missing longitudinal data; (6) absence of interventional data; (7) temporal heterogeneity over 3.5 years; (8) ethnic/surgical homogeneity | Yes |
| 20 | Give a cautious overall interpretation of results considering objectives, limitations, multiplicity of analyses, and other relevant studies. | Discussion §4 — findings framed as pragmatic GLIM operationalisation vs PNI, not GLIM framework per se; exploratory/confirmatory distinction maintained; GLIM null findings contextualised in domain-specific pathophysiology; PNI findings consistent with external meta-analysis | Yes |
| 21 | Discuss the generalisability (external validity) of the study results. | Limitations §4 paragraph 1 — single Chinese tertiary academic centre; lower malnutrition prevalence (6.5–6.9%) vs Western cohorts (15–30%); ethnic homogeneity; predominantly elective degenerative surgery (84.9%); limited emergency, trauma, and oncologic cases | Yes |
| **OTHER INFORMATION** | | | |
| 22 | Give the source of funding and the role of the funders for the present study and, if applicable, for the original study on which the present article is based. | Funding Statement — "None to declare"; no external funding received; Authors' Contribution statement revised to remove "Funding Acquisition" role per reviewer comment | Yes |

** Give information separately for cases and controls in case-control studies and, if applicable, for exposed and unexposed groups in cohort and cross-sectional studies.*

*Note: An Explanation and Elaboration article discusses each checklist item and gives methodological background and published examples of transparent reporting. The STROBE checklist is best used in conjunction with this article (freely available on the Web sites of PLoS Medicine at http://www.plosmedicine.org/, Annals of Internal Medicine at http://www.annals.org/, and Epidemiology at http://www.epidem.com/). Information on the STROBE Initiative is available at www.strobe-statement.org.*
